# Metapopulation distribution shapes year‐round overlap with fisheries for a circumpolar seabird

**DOI:** 10.1002/eap.70019

**Published:** 2025-04-21

**Authors:** Kalinka Rexer‐Huber, Thomas A. Clay, Paulo Catry, Igor Debski, Graham Parker, Raül Ramos, Bruce C. Robertson, Peter G. Ryan, Paul M. Sagar, Andrew Stanworth, David R. Thompson, Geoffrey N. Tuck, Henri Weimerskirch, Richard A. Phillips

**Affiliations:** ^1^ Department of Zoology University of Otago Dunedin Otago New Zealand; ^2^ Parker Conservation Dunedin Otago New Zealand; ^3^ Institute of Marine Sciences, University of California Santa Cruz Santa Cruz California USA; ^4^ People and Nature, Environmental Defense Fund Monterey California USA; ^5^ Marine and Environmental Sciences Centre (MARE)/ARNET – Aquatic Research Network, Ispa – Instituto Universitário Lisbon Portugal; ^6^ Aquatic Unit, Department of Conservation Wellington New Zealand; ^7^ Departament de Biologia Evolutiva, Ecologia i Ciències Ambientals, Facultat de Biologia Universitat de Barcelona Barcelona Spain; ^8^ Institut de Recerca de la Biodiversitat (IRBio), Universitat de Barcelona Barcelona Spain; ^9^ FitzPatrick Institute of African Ornithology University of Cape Town Rondebosch Western Cape South Africa; ^10^ National Institute of Water and Atmospheric Research Ltd. Wellington New Zealand; ^11^ Falklands Conservation, Stanley Falkland Islands UK; ^12^ The Commonwealth Scientific and Industrial Research Organization (CSIRO) Environment Business Unit Hobart Tasmania Australia; ^13^ Centre d'Étude Biologique de Chizé, UMR 7273 CNRS – Université de La Rochelle Villiers‐en‐Bois France; ^14^ British Antarctic Survey, Natural Environment Research Council Cambridge Cambridgeshire UK

**Keywords:** biologging, bycatch mitigation, geolocator, longline fisheries, migratory connectivity, Regional Fisheries Management Organization, trawl fisheries, white‐chinned petrel

## Abstract

Although fisheries bycatch is the greatest threat to many migratory marine megafauna, it remains unclear how population exposure to bycatch varies across the global range of threatened species. Such assessments across multiple populations are crucial for understanding variation in impacts and for identifying the management bodies responsible for reducing bycatch. Here, we combine extensive biologging data from white‐chinned petrel (*Procellaria aequinoctialis*) populations (representing >98% of their global breeding population) with pelagic and demersal longline and trawl fishing effort to map the global distribution and fisheries‐overlap hotspots for the most bycaught seabird in the Southern Hemisphere. We tracked the year‐round movements of 132 adults in 2006–2018 and examined spatial overlap among seven populations comprising three genetically distinct groupings (metapopulations). Foraging areas during the nonbreeding season were more concentrated than during breeding, with birds from all populations migrating to continental shelf or upwelling zones, but with low spatial overlap among metapopulations. Fisheries overlap differed more among than within metapopulations, underlining that these should be considered separate management units. Overlap with pelagic longline fisheries was greatest for Indian Ocean populations, and from the fleets of South Africa, Japan, Taiwan, and Spain, off southern Africa and in the High Seas. Overlap with demersal longline and trawl fisheries was greatest for Indian and Atlantic Ocean populations, within the Exclusive Economic Zones of South Africa, Namibia, and Argentina, and with the South Korean demersal longline fleet in the High Seas. The high overlap with South Korean longliners in the southwest Atlantic Ocean is of particular concern as demersal fishing in this region is not covered by any Regional Fisheries Management Organization (RFMO). We also identified fisheries‐overlap hotspots within RFMOs where there are no seabird‐bycatch mitigation requirements (1.5%–53.1% of total overlap within the area of competence of each RFMO), or where current mitigation regulations need to be strengthened. Our recommendations are that management bodies target the high‐priority fisheries we have identified for improved bycatch monitoring, mandatory best‐practice bycatch mitigation, and close monitoring of compliance, given the conservation concerns for white‐chinned petrels and other threatened seabirds.

## INTRODUCTION

Migratory connectivity describes the geographical linkages between populations and has important implications for conservation because it determines co‐occurrence across the annual cycle and hence relative exposure to threats (Martin et al., 2007; Runge et al., 2014; Webster et al., 2002). Low (or weak) migratory connectivity occurs when individuals from a particular breeding population spread over a large nonbreeding area and mix with individuals from other breeding populations (Cohen et al., 2018; Finch et al., 2017). Reducing threats in areas of inter‐population mixing may benefit widely separated breeding populations (e.g., Studds et al., 2017). In contrast, under a scenario of high (strong) migratory connectivity, individuals use discrete, population‐specific nonbreeding areas, which likely have a distinct set of localized threats; as such, management in these areas can target threats to specific populations (Dunn et al., 2019; Oppel et al., 2021; Runge et al., 2014).

The wide‐ranging and transboundary movements of marine megafauna, such as seabirds, place them at risk from multiple threats within national waters and in the High Seas (areas beyond national jurisdiction) leading to diverse management challenges (Beal et al., 2021; Ramos et al., 2013). Fisheries bycatch—the mortality of nontarget species through hooking or entanglement in lines, collisions with trawl warps and monitoring cables, or capture in nets—is the most immediate threat to many seabird populations (Dias et al., 2019; Lewison et al., 2004). Seabirds are particularly susceptible due to their longevity, delayed maturity, and low reproductive rates, and because their high mobility leads them to encounter multiple fishery types and fleets with variable use of bycatch mitigation measures (Pardo et al., 2017; Phillips et al., 2016). Since the late 1980s, there have been concerted global efforts to monitor and mitigate seabird bycatch, with some impressive successes, including >90% reductions in certain fisheries (Da Rocha et al., 2021; Phillips et al., 2016). However, gaps remain in terms of monitoring compliance with mitigation requirements and understanding when and where bycatch occurs because of low observer coverage and engagement by fishing fleets and Illegal, Unreported, and Unregulated (IUU) fishing (Anderson et al., 2011; Phillips et al., 2016; Tuck et al., 2003).

Appropriate management of seabird‐fisheries interactions requires a robust understanding of their spatiotemporal dynamics. Recent developments in biologging (GPS and radar loggers) and vessel tracking using Vessel Monitoring Systems (VMS) and Automatic Identification Systems (AIS) have facilitated the study of factors influencing seabird attraction to and interaction with fishing vessels at fine scales (Banda et al., 2024; Carneiro et al., 2022; Orben et al., 2021; Weimerskirch et al., 2020). However, these analyses are usually restricted to a particular geographic region, breeding population, life‐history stage, or fishery, due to logistical challenges and political or commercial sensitivities in releasing vessel locations. As seabird and fishing activity are unevenly distributed and often vary over large (ocean‐basin) scales, assessing the overlap of multiple populations with fishing fleets across jurisdictions provides a more holistic perspective on the threats to a species (Clay et al., 2019b; Delord et al., 2014). Moreover, bycatch can have disproportionate impacts on particular populations, so standardized assessments of relative overlap with fisheries are crucial for understanding species‐wide variation in exposure to bycatch risk, links with demography, and the fishing fleets responsible (Baetscher et al., 2022; Corbeau et al., 2021; Genovart et al., 2018).

The white‐chinned petrel (*Procellaria aequinoctialis*) is the seabird most commonly caught as bycatch in the Southern Hemisphere (Anderson et al., 2011; Phillips et al., 2006; Rollinson et al., 2017). Although highly abundant (~1.1 million breeding pairs globally; Phillips et al., 2016), the species is listed as Vulnerable by the IUCN as monitored populations are mostly in decline (Berrow et al., 2000; Nel et al., 2002; but see Dasnon et al., 2022). White‐chinned petrels range from the subtropics south to Antarctica, and because they forage in biologically productive regions, they interact with and are caught in a wide variety of fisheries, predominantly pelagic and demersal longline and trawl fleets (Delord et al., 2010; Phillips et al., 2006; Weimerskirch et al., 1999) (reviewed in Appendix S1: Table S1). They are opportunistic scavengers, attracted to vessels to feed on baited hooks or discards, and their competitive nature, maneuverability, ability to dive deeper (over 20 m) than competitors such as albatrosses, and nocturnal activity increase their likelihood of capture (Frankish et al., 2021; Jiménez et al., 2012; Mackley et al., 2011).

**FIGURE 1:**
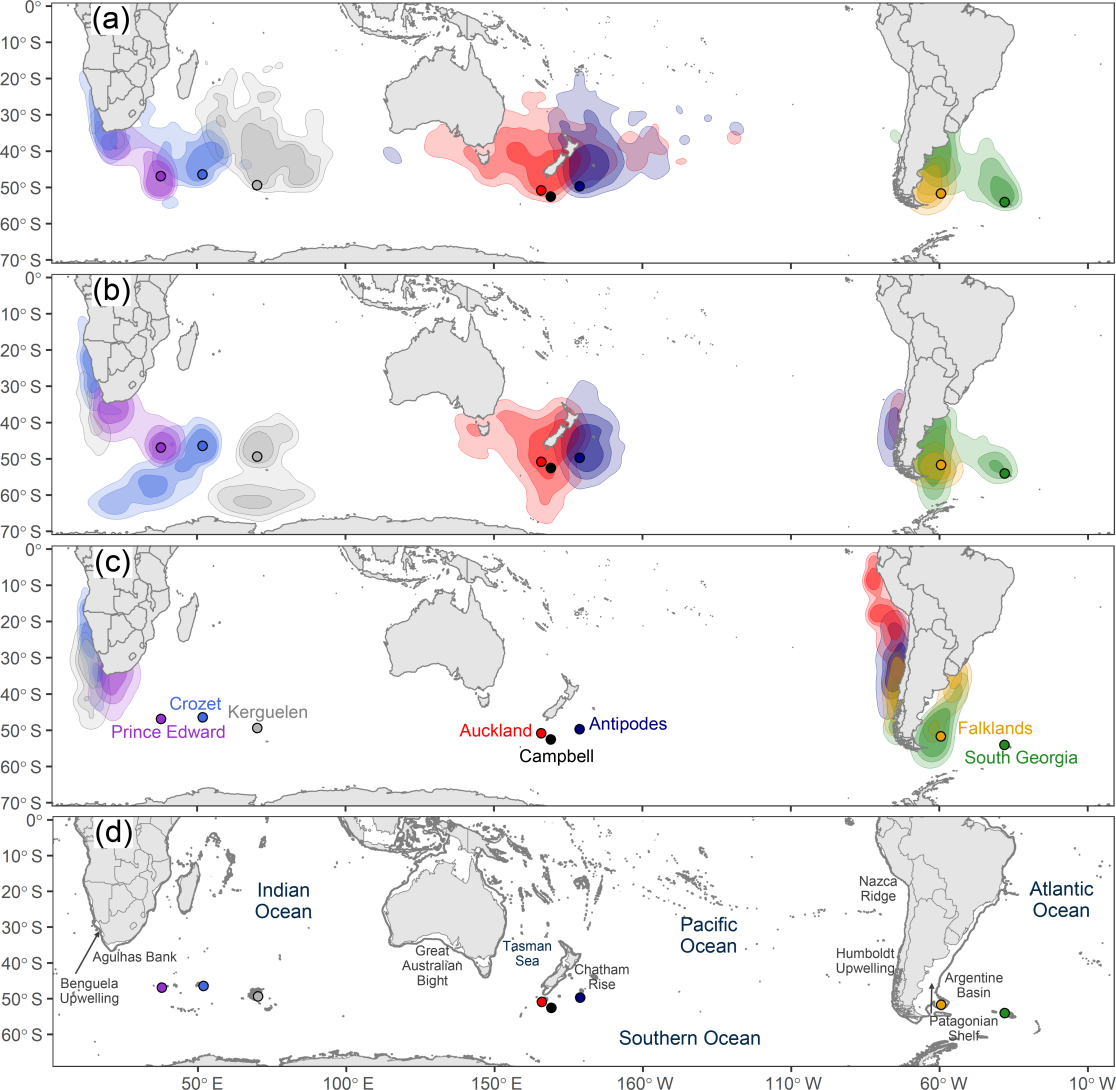
Utilization distributions (UDs) of white‐chinned petrels (*Procellaria aequinoctialis*) tracked with geolocators from different island populations over the annual cycle: (a) prelaying (October–November), (b) breeding (December–April), and (c) nonbreeding (May–September). The 30%, 50%, and 70% UD contours are colored in progressively lighter shades for each tracked population. (d) Map indicates the location of oceanographic or topographic features named in text, with the 200‐m isobath shown by a dark gray line.

White‐chinned petrels breed in eight subantarctic island groups (Table 1, Figure 1) and group into three genetic units according to ocean basin (hereafter, Indian, Pacific, and Atlantic metapopulations), along with some differences among populations within the Atlantic and Pacific Oceans (Rexer‐Huber et al., 2019; Techow et al., 2009, 2016). Four widely separated populations in the southwest Indian Ocean and South Georgia have been tracked before, allowing high‐risk fisheries‐overlap areas to be identified (Clay et al., 2019b; Delord et al., 2010; Frankish et al., 2021; Péron et al., 2010; Phillips et al., 2006; Rollinson et al., 2018; Weimerskirch et al., 1999). The distributions of white‐chinned petrel populations from New Zealand and the Falkland Islands are hitherto unknown. Given that migration strategies appear to drive genetic differentiation in some seabirds (Friesen et al., 2007; Rayner et al., 2011), we might expect divergent nonbreeding distributions across metapopulations, with implications for conservation if genetic differences align with differential fisheries overlap metrics.

**TABLE 1:** Details of tracking (geolocator) data analyzed in this study, and population sizes and trends of white‐chinned petrels (*Procellaria aequinoctialis*).

Population (jurisdiction)	No. individuals		Years tracked	Mean days tracked	Validated locations	Annual breeding pairs	% breeding Pop.	Pop. trend
	Retrieved (deployed)	Usable files						
Prince Edward (South Africa)	12 (21)	12	2009–2013	870	14,079	35,685^a^	2.7	↑^i^
Crozet (France)	14 (20)	10	2007–2008	355	3725	23,600^b^	1.8	↑^j^
Kerguelen (France)	27 (30)	13	2006–2008	337	4707	234,000^c^	18.0	?
Auckland (New Zealand)	43 (62)	37	2013–2018	315	13,707	184,000^d^	14.2	?
Antipodes (New Zealand)	30 (34)	22	2008–2010	329	8126	26,400^e^	2.0	?
Campbell (New Zealand)	…	…	…	…	…	22,000^f^	1.7	?
Falklands^l^ (United Kingdom)	16 (27)	14	2014–2015	391	6075	200^g^	<0.1	?
South Georgia (United Kingdom)	26 (37)	24	2013–2015	308	7517	773,150^h^	59.5	↓^k^
Total	168 (231)	132	2006–2018	415	57,936	1,277,035	98.3	…

^a^ Dilley et al. (2019), occupancy from Ryan et al. (2012).

^b^ Barbraud et al. (2008).

^c^ Barbraud et al. (2009).

^d^ Rexer‐Huber et al. (2017, 2020).

^e^ Rexer‐Huber et al. (2023).

^f^ Rexer‐Huber et al. (2017).

^g^ Reid et al. (2007) plus Rexer‐Huber unpublished data.

^h^ Martin et al. (2009), removing correction for the proportion that did not attempt to breed for consistency with other estimates.

^i^ Dilley et al. (2017).

^j^ Dasnon et al. (2022).

^k^ Berrow et al. (2000).

^l^ Birds were tracked from Kidney Island and New Island, but the data were pooled as distributions were similar (see Appendix S2: Section S1 for details).

Here, we combined geolocator (Global Location Sensor, GLS) datasets from white‐chinned petrels tracked from seven populations representing >98% of global breeders and fishing effort data to determine how year‐round population distributions influence exposure to bycatch from industrial longline and trawl fishing fleets. Our aims were to (1) quantify spatial overlap and migratory connectivity across the annual cycle among and within metapopulations, and (2) combine white‐chinned petrel distributions and fishing effort to determine where overlap with fisheries, and by inference bycatch risk, is likely to be highest, and to determine variation according to population, season, and flag state. We then calculated fisheries overlap within Exclusive Economic Zones (EEZs; 200 nautical miles from the shore) and in the High Seas within the areas of each Regional Fisheries Management Organization (RFMO) and the Commission for the Conservation of Antarctic Living Marine Resources (CCAMLR) to (3) identify political responsibilities for monitoring of bycatch rates, regulation, and enforcement of bycatch mitigation. Since some populations are larger than others by several orders of magnitude, we weighted distributions to (4) map the global density of breeding adult white‐chinned petrels and calculate the contribution of each population to regions with elevated fisheries overlap (fisheries‐overlap hotspots). Lastly, we considered the seabird bycatch mitigation measures mandated by each RFMO, using the geographic boundaries of mandated measures to (5) examine the degree to which fisheries‐overlap hotspot areas are covered by bycatch regulations.

## METHODS

### Device deployment and data processing

White‐chinned petrels were tracked from seven major populations: Marion Island, Prince Edward Islands (37°51′ E, 46°52′ S), Crozet (51°51′ E, 46°26′ S), and Kerguelen (70°13′ E, 49°21′ S) in the southwest Indian Ocean; Auckland (165°56′ E, 50°50′ S) and Antipodes (178°48′ E, 49°40′ S) Islands in the southwest Pacific Ocean; and the Falkland Islands (59°31′ W, 51°41′ S) and Bird Island, South Georgia (38°03′ W, 54°00′ S) in the southwest Atlantic Ocean (Table 1). Breeding birds were removed from burrows, and geolocators (MK4, 9 and 13, British Antarctic Survey, Cambridge UK; MK3005, Biotrack, Wareham UK; Intigeo C240, Migrate Technology, Cambridge UK) were attached with cable ties to leg rings. The logger plus attachment materials (2.6–3.0 g) represented <0.3% of bird mass. Loggers were ground‐truthed at the colony for ~7 days (range 3–25 days) before deployment and after retrieval.

Raw light data from all loggers were processed by KRH for consistency using TransEdit2 and IntiProc software. Sunset and sunrise times were determined at a fixed threshold (12) in the light curves, and two positions per day were estimated using the sun elevation angle of best fit (mean of 4–7°), determined for each set of devices. During processing, all light curves were inspected, identifying light‐level interference as any obviously interrupted or unrealistic curves. Locations were then filtered in r v.4.0.4 (R Core Team, 2022) to remove those that were south of 70° S and north of 0°, within 15 days of the equinoxes or that required unrealistic flight speeds (>116 km/h; Péron et al., 2010; Weimerskirch et al., 1999). Many of the loggers did not record temperature, so latitudes were estimated based on light data only.

### Population distributions and global densities

Spatial analyses and data visualization used r v.4.1.2 (R Core Team, 2022). We first conducted a representativeness test which revealed that the sample of tracked birds was sufficient to represent the space use of each population (see Appendix S2: Section S2 for details). For each month, kernel Utilization Distributions (UDs) were calculated using the package adehabitatHR (Calenge, 2006). Locations were first projected to a Lambert Equal Area projection centered around the median longitude and latitude of each population in each month, and UDs were created using a 10‐km^2^ grid cell and smoothing (*h*) of 186 km, corresponding to the average GLS error (Phillips et al., 2004). Monthly UDs were created for all individuals with at least five locations, and then summed across individuals to create a population‐level grid. Although for most months the median number of locations per individual was >20, this threshold allowed us to generate UDs for September when there were only 5–10 locations per individual. There were insufficient locations to create UDs for March.

Monthly distribution grids were then scaled so all cells summed to a value of one and were resampled to a 5° resolution or to a 2° resolution for use in different fisheries‐overlap analyses (see below). We averaged monthly grids corresponding to three major periods: prelaying (October–November), breeding (December–April), and nonbreeding (May–September). The periods broadly matched with annual breeding cycles based on patterns of burrow attendance (from light data) and large‐scale movements indicating the start and end of migration. Given populations only differed in the mean date of first return to the burrow (range: 2 October–6 November, *F*_6,102_ = 5.7, *p* < 0.001), we applied the same chronology to all populations. UD contours were calculated at three isopleth levels: 30% (core areas, high probability of use), 50% (home range, intermediate use), and 70% (general use; Figure 1).

**FIGURE 2:**
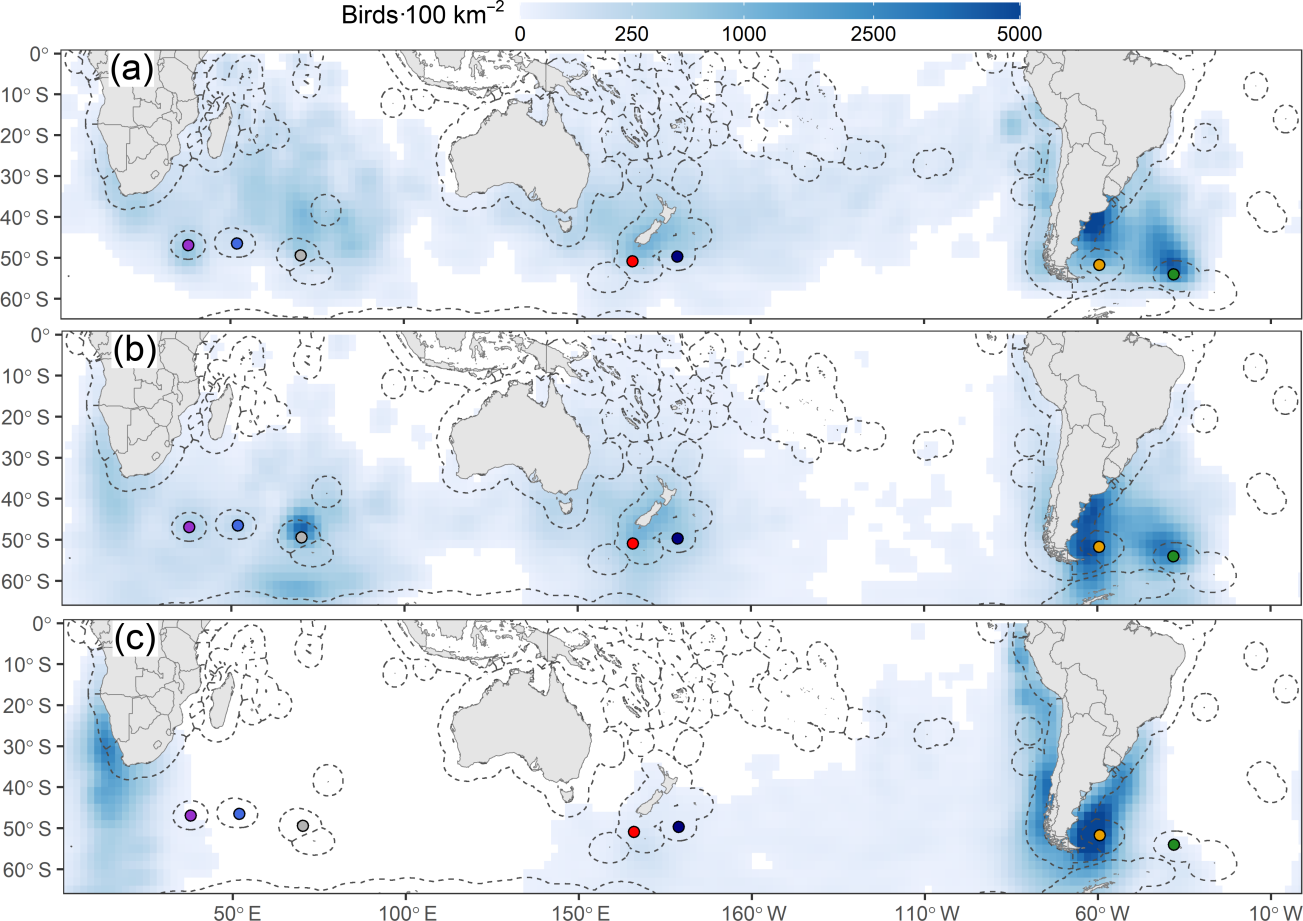
Predicted densities of adult white‐chinned petrels (*Procellaria aequinoctialis*) (birds 100 km^−2^) tracked with geolocators during (a) prelaying (October–November), (b) breeding (December–April) and (c) nonbreeding (May–September). Densities are based on at‐sea distributions resampled to a 2° resolution and weighted by population sizes (Table 1; breeding pairs multiplied by two). Populations: Prince Edward (purple), Crozet (royal blue), Kerguelen (gray), Auckland (red), Antipodes (navy blue), Falklands (yellow) and South Georgia (green). Exclusive Economic Zone boundaries are shown by gray dashed lines. Only cells with densities >1 bird 100 km^−2^ are shown.

Spatial overlap among all pairs of the seven white‐chinned petrel populations was quantified using Bhattacharya's Affinity (BA) overlap index in each period using adehabitatHR (Calenge, 2006). BA values range between 0 (no overlap) and 1 (complete overlap). Overlap scores among and within metapopulations were compared for each period using Wilcoxon tests. We quantified migratory connectivity by comparing distance matrices of individual breeding and nonbreeding locations using Mantel correlations (Cohen et al., 2018) (see Appendix S2: Section S3 for details). Correlations were calculated across all individuals and then separately within metapopulations. To map the global density of breeding adult white‐chinned petrels during each period, we resampled population distributions to a 2° spatial resolution, multiplied by the total number of breeding pairs multiplied by two, and then summed across populations. The number of birds in each grid cell was divided by its area to estimate the number of breeding adults per 100 km^2^ (Figure 2).

### Fisheries data

Fishing effort data were obtained from two sources: (1) monthly logbook effort at a 5° resolution reported to RFMOs, CCAMLR, or national fisheries agencies, and (2) daily satellite AIS data from Global Fishing Watch (GFW). Our analyses included logbook data for 2000–2009, as later datasets were not made available by some fisheries bodies, presumably due to political or commercial sensitivities. We used the GFW v.2 dataset at a 0.01° resolution for 2012–2020, which we resampled to both a 5° and monthly resolution for comparison with logbook data and a 2° and monthly resolution to assign regulatory responsibility and to identify fisheries‐overlap hotspots at the species level. GFW combines public vessel registries and machine learning to identify fishing vessels and detect when they are actively fishing (Kroodsma et al., 2018). For more information on fisheries datasets, see Appendix S2: Section S4. As neither dataset covered the full temporal range of the white‐chinned petrel tracking data (2006–2018, Table 1), and both have known biases, we used both fisheries datasets for a complementary view of the overlap of white‐chinned petrels with fishing fleets and gear types. Logbook data show total effort at a coarse resolution and are often incomplete, so estimates have to be raised or extrapolated based on catch records (Tuck et al., 2003). AIS data from GFW provide vessel information at a finer spatiotemporal resolution, but only 50%–70% of vessels >24 m are fitted with and transmit AIS, varying strongly by region (Kroodsma et al., 2018; Welch et al., 2022). Regardless, maps of effort based on logbook and AIS data (at 5° and 2° resolutions) revealed similar regions of elevated fishing activity, and both datasets for pelagic longline fisheries showed high concordance (Appendix S2: Section S5).

### Population‐specific fisheries overlap and global hotspots

We calculated monthly fisheries overlap per grid cell—an index of potential bycatch risk—as the time spent by white‐chinned petrels multiplied by the number of hooks or fishing hours (e.g., Carneiro et al., 2020; Jiménez et al., 2016). Before doing so, we assigned all overlap values corresponding to distribution grid cells representing the 90th percentile of UDs to zero to reduce the potential influence of cells with high fishing effort but low usage by petrels, particularly at the edge of their distribution where geolocation error often leads to range inflation. Fisheries overlap was calculated separately for each dataset (AIS and logbook), gear type, and flag state at a 5° resolution. Important fleets were considered to be flag states contributing to at least 5% of total overlap for that population and gear type (Appendix S3).

We calculated the overlap between both population‐level and species‐level distributions and AIS effort data at a 2° resolution to assign responsibility in terms of fisheries jurisdiction and to identify fisheries‐overlap hotspots at the species level. Monthly population distribution grids were first overlaid on shapefiles of country land borders and EEZs (v. 11; from https://www.marineregions.org/downloads.php) and RFMO and CCAMLR borders (from http://fao.org/geonetwork) to estimate the annual time spent by each population in each jurisdiction. Here, national jurisdictions are the EEZs of each country, including overseas dependencies and disputed territories (Beal et al., 2021). We then overlaid monthly population‐level overlap grids with EEZ, RFMO, and CCAMLR boundaries to determine overlap in EEZs versus overlap in areas of competency of the RFMOs and CCAMLR within the High Seas. For pelagic longline fisheries, we selected RFMOs responsible for the management of tuna and billfish (Scombridae): International Commission for the Conservation of Atlantic Tunas (ICCAT), Indian Ocean Tuna Commission (IOTC), Western and Central Pacific Fisheries Commission (WCPFC) and Inter‐American Tropical Tuna Commission (IATTC). For demersal longline and trawl fisheries, we selected CCAMLR and RFMOs responsible for nontuna fisheries (mainly finfish and squid): South East Atlantic Fisheries Organization (SEAFO), Southern Indian Ocean Fisheries Agreement (SIOFA) and South Pacific Regional Fisheries Management Organization (SPRFMO). As nontuna RFMOs are only responsible for High Seas fishing areas, we only considered areas of fisheries overlap within the High Seas RFMOs and CCAMLR for consistency among fishing gear types. Following Beal et al. (2021), we did not separately examine overlap with fishing effort data from the Commission for the Conservation of Southern Bluefin Tuna (CCSBT) as this RFMO has competency over a species rather than a region, and the effort data are also reported to the other four tuna RFMOs. We counted overlap in areas covered by two RFMOs (e.g., IATTC and WCPFC) separately for each RFMO.

To identify fisheries‐overlap hotspots at the species level, we weighted fisheries‐overlap grids for each population according to the proportion of estimated global numbers breeding at each island group and summed those to generate three species‐level grids, one for each gear type (pelagic longline, demersal longline, and trawl). We defined fisheries‐overlap hotspots as cells with the top 25% of overlap scores and split these into five regions based on longitudinal boundaries, which were selected either to match the areas of competence of RFMOs or to visually separate distinct clusters of high‐overlap values: southeast Pacific Ocean (150° W–70° W), southwest Atlantic Ocean (70° W–20° W), southern Africa (20° W–45° E), Indian Ocean (45° E–120° E) and southwest Pacific Ocean (120° E–150° W). Within each fisheries‐overlap hotspot region, the percentage of total overlap represented by each population is a proxy for its potential contribution to bycatch. To visualize fisheries overlap across the three gear types combined, we scaled each overlap grid to one, summed across grids, and then divided values in each cell by three to create a combined fisheries‐overlap grid. We note that the maximum values of overlap with gear type are unlikely to be equivalent, but this approach allows us to identify regions of high overlap across gear types. For gear‐specific maps, see Appendix S3: Figure S1.

Lastly, we examined the degree to which pelagic longline fisheries that overlap with hotspots for white‐chinned petrels were covered by seabird bycatch mitigation regulations mandated by RFMOs. We split RFMO regions according to the spatial boundaries of regulations, which were summarized into the following three mitigation categories: (1) no mitigation measures required, (2) one measure required, and (3) two measures required. The measures generally include a combination of weighted branch lines, night setting, and tori (bird‐scaring or streamer) lines, or hook‐shielding devices (see Appendix S2: Section S6 for details). Monthly species‐level fisheries overlap grids were cropped by RFMO shapefiles, and the percentage of total overlap in each RFMO and mitigation category was summed across the year. All means are provided ± SD unless stated otherwise.

## RESULTS

Overall, 168 geolocators were retrieved (73% of 231 deployed; Table 1); of these, 36 (21%) failed to record for >30 days, or the data were unusable because of light‐level interference caused by shading or other issues. In total, 132 white‐chinned petrels were tracked for 375 ± 212 days, including 10 birds tracked for ~1000 days.

### Distributions and global density patterns

During prelaying (October–November), white‐chinned petrels foraged over large areas (core areas: 1.3 ± 0.7 million km^2^) to the north of their colonies (~30–55° S), particularly the Benguela Upwelling, southwest Indian Ocean, Great Australian Bight, Tasman Sea, Chatham Rise, and further east, Patagonian Shelf and Argentine Basin (Figure 1a). During breeding months (December–April), core areas extended to higher latitudes (20–65° S; Figure 1b) and over large areas (1.1 ± 0.4 million km^2^). During the nonbreeding season (May–September), core areas were smaller than in other periods (Figure 1c) and were in the Benguela Upwelling, Agulhas Bank, Humboldt Upwelling, and Patagonian Shelf. Globally, the highest white‐chinned petrel densities were north of South Georgia and on the central Patagonian Shelf during prelaying, in similar areas and also north of Kerguelen during breeding, and on the Patagonian Shelf, Benguela Upwelling, and Humboldt Upwelling during nonbreeding (Figure 2). On the southern Patagonian Shelf, white‐chinned petrel densities reached >5000 birds 100 km^−2^.

### Spatial overlap and migratory connectivity

Spatial overlap of individuals was higher within than among the three metapopulations across the annual cycle (Wilcoxon rank‐sum test based on 50% UDs; prelaying: *p* < 0.001 breeding: *p* < 0.001: nonbreeding: *p* = 0.001) (Appendix S2: Table S1). Migratory connectivity was moderate to high across all populations (Mantel correlation coefficient [*r*_M_] = 0.64, *p* = 0.002) and low within metapopulations (Atlantic: *r*_M_ = 0.08, *p* = 0.040; Pacific: *r*_M_ = 0.19, *p* = 0.004; Indian: *r*_M_ = 0.20, *p* = 0.005), indicating that white‐chinned petrel distributions were more clustered among than within metapopulations. All populations overlapped with at least one other, though overlap was generally low to moderate within each metapopulation, particularly during breeding (Figure 1; Appendix S2: Table S1). The only overlap of populations from different oceans was during nonbreeding; >50% of birds from both Atlantic populations migrated to the Humboldt Upwelling and overlapped with Antipodes birds.

### Population‐specific overlap with fisheries

**FIGURE 3:**
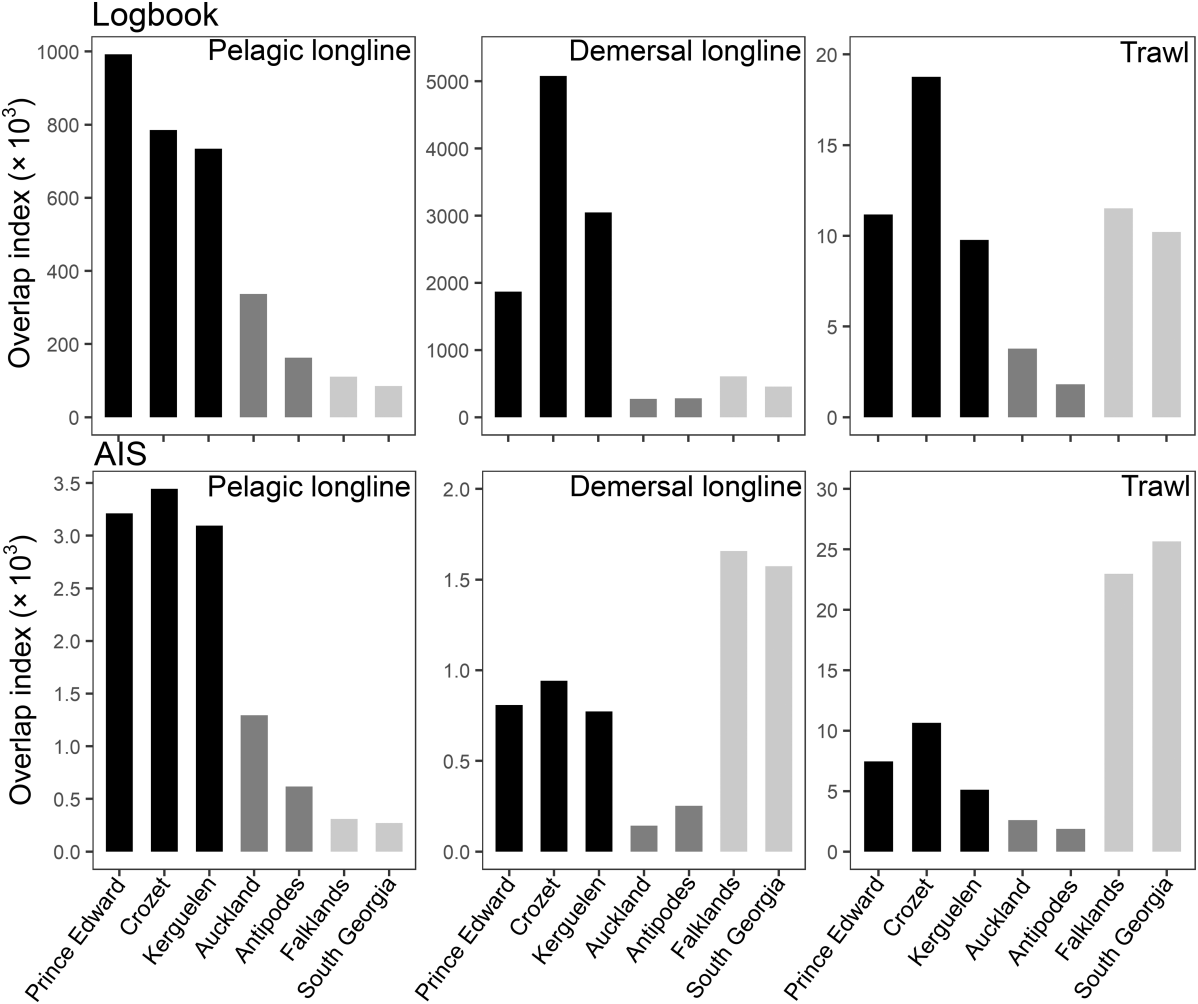
Year‐round fisheries overlap for white‐chinned petrel (*Procellaria aequinoctialis*) populations based on vessel logbooks (top row) and Automatic Identification Systems (AIS) (bottom row) at a 5° resolution. Overlap indices were summed for each gear type across all months and divided by 10^3^, and were based on fishing hooks set for logbook pelagic and demersal longline datasets or fishing hours for logbook trawl and all AIS datasets. Shading corresponds to ocean‐basin grouping: Indian Ocean (black), Pacific Ocean (dark gray), and Atlantic Ocean (light gray).

**FIGURE 4:**
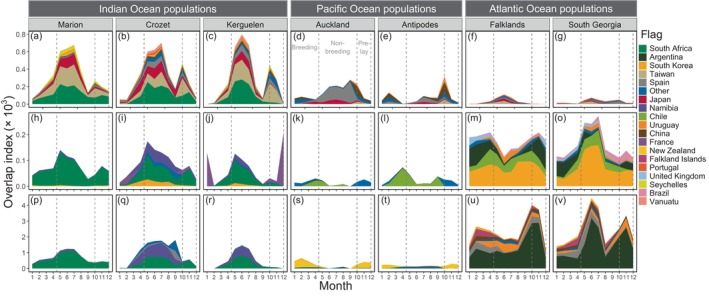
Monthly overlap of white‐chinned petrel (*Procellaria aequinoctialis*) populations with pelagic longline (top row), demersal longline (middle row), and trawl (bottom row) fishing effort, according to flag state. Overlap was conducted with vessel Automatic Identification Systems effort at a 5° spatial resolution (see Appendix S3: Figure S2 for overlap based on logbook effort). Flag states were assigned the same color across gear types (rows). Dashed vertical lines indicate the timing of breeding, nonbreeding, and prelaying periods.

Overlap with fisheries varied considerably among populations but was for the most part more similar within than among metapopulations at the scale of ocean basins (Figure 3). Although there were differences between the fishing effort datasets (AIS vs. logbook), the regions identified as having the highest fisheries overlap were remarkably similar (Figure 4; Appendix S3: Figure S3). Overlap with pelagic longline fisheries was greatest for white‐chinned petrel populations breeding in the Indian Ocean and least for those from the Atlantic Ocean, regardless of data source (Figure 4; Appendix S3: Figure S2). Pacific populations had the lowest overlap with trawl and demersal longline fisheries. Indian Ocean populations mostly had higher overlap with demersal and trawl fisheries than Atlantic populations based on logbook data, although AIS data showed the opposite pattern (Figure 3).

Overlap of white‐chinned petrels with pelagic longline effort was highest during May–August (nonbreeding), with another peak of October–November (prelaying exodus) for all but the Atlantic populations (Figure 4a–g). The important flag states based on logbook data were Taiwan, Japan, Spain, South Africa, and Namibia (representing 89.4% of overlap), and based on AIS data were South Africa, Taiwan, Japan, and Spain (79.3%; Appendix S3: Tables S1–S3). Auckland birds overlapped more with pelagic longline fisheries (Japan, Spain) than Antipodes birds, probably due to their more northerly subtropical distribution (Figures 1c and 2). Overlap of Indian Ocean populations with demersal longline and trawl fisheries (mainly South African and Namibian flagged vessels) was highest during nonbreeding months off southern Africa (Figure 4h–j,p–r). There was also overlap for Kerguelen and Crozet birds with French demersal longliners around their colonies (Figure 4i–j). For the Pacific populations, overlap with demersal longline and trawl fisheries mostly involved New Zealand vessels during prelaying and breeding (Figure 4k–l,s–t), generally corresponding well with the months when observed captures by these fleets were highest (see Appendix S4). Lastly, there was substantial year‐round overlap (based on AIS data) of the Atlantic populations with South Korean, Chilean, and Argentine demersal longline vessels and Argentine, Spanish, and Uruguayan trawl vessels (Figure 4m–o,u–v). There was a peak in overlap during summer for Falklands birds, and during winter for South Georgia birds, both with South Korean longliners and Argentine trawlers operating around the Patagonian Shelf. In contrast, logbook data indicated low overlap for the two Atlantic populations with Argentine longliners, while the peak in overlap with trawlers generally occurred during late breeding (Appendix S3: Figure S2).

### Fisheries‐overlap hotspots and regulatory responsibility

**FIGURE 5:**
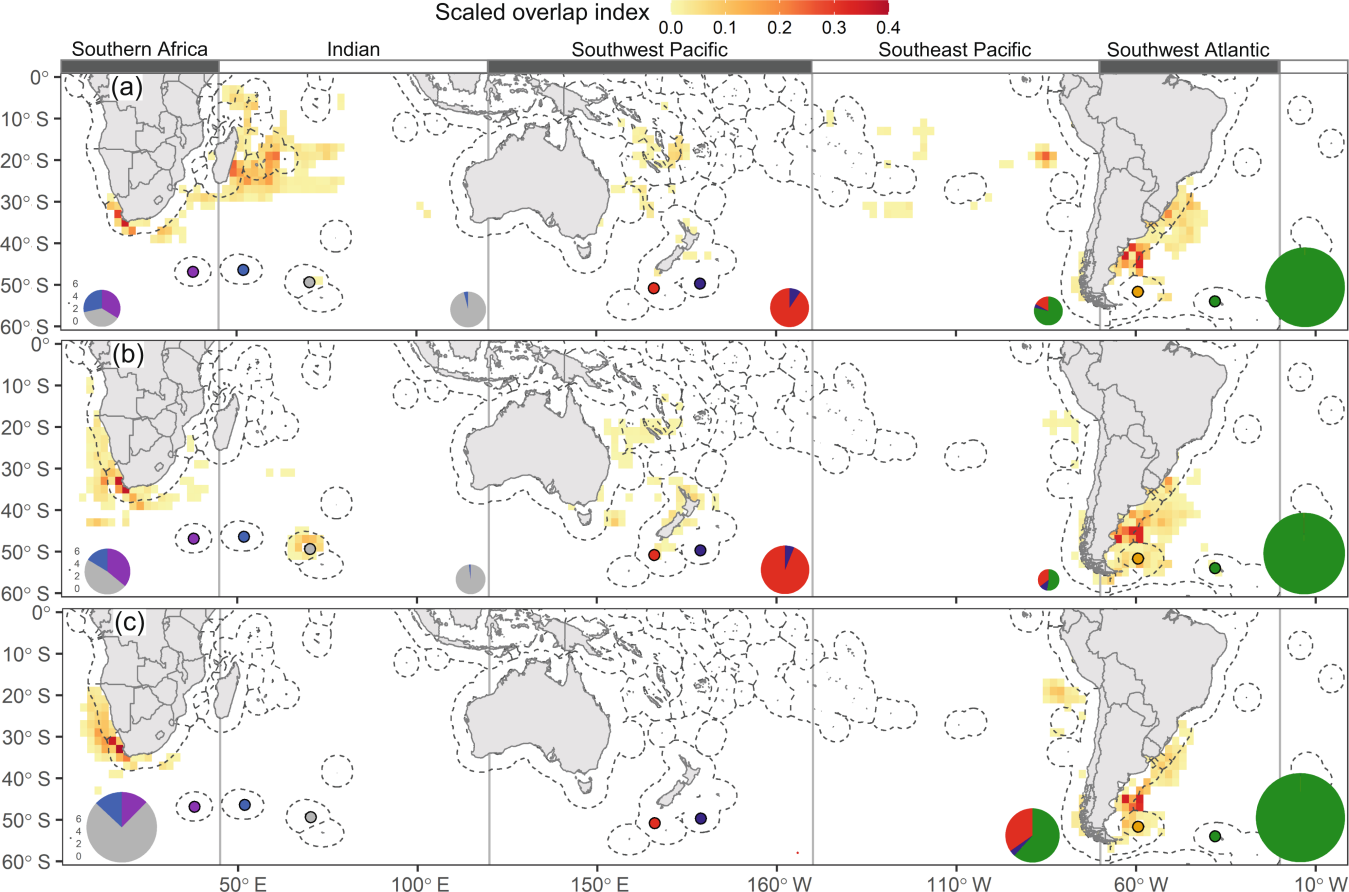
Species‐level fisheries‐overlap hotspots for white‐chinned petrels (*Procellaria aequinoctialis*) and the contribution of each population to total overlap, during (a) prelaying (October–November), (b) breeding (December–April), and (c) nonbreeding (May–September). Fisheries overlap was calculated for each population and gear type, weighted by population size, summed across gear types, and scaled to a maximum index value of 1. Five fisheries‐overlap hotspot regions were identified (indicated by longitudinal gray lines) and the proportion represented by each population is shown by a pie chart, with the size scaled for each region according to the total species‐level fisheries overlap. Numbers on the bottom left pie chart indicate the log of the total overlap in each region. Boundaries of Exclusive Economic Zones are shown by dashed gray lines.

Hotspots of fisheries overlap were identified in the southwest Atlantic (Patagonian Shelf and further offshore), off southern Africa, and to a lesser extent off Chile and Peru, and in the southwest Indian and Pacific Oceans (Figure 5; Appendix S3: Figures S1 and S3). Fisheries overlap off southern Africa was high for several white‐chinned petrel populations, although the contribution of each population varied according to the season; for example, a greater percentage of the total overlap off southern Africa comprised birds from Kerguelen during nonbreeding (74.6%) than at other times (prelaying = 37.8%, breeding = 47.7%; Figure 5). Other fisheries‐overlap hotspots included waters around Kerguelen and New Zealand during breeding months, and from the Humboldt Upwelling up to the Nazca Ridge in the southeast Pacific during prelaying (for Antipodes, Auckland, and South Georgia birds; Figure 5a,b).

**FIGURE 6:**
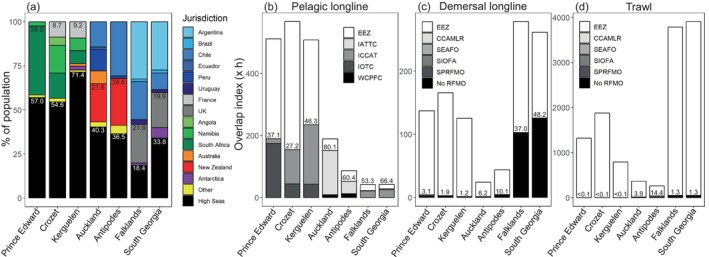
(a) Proportion of time spent by white‐chinned petrel (*Procellaria aequinoctialis*) populations and (b–d) fisheries overlap in national jurisdictions versus the High Seas. (a) Jurisdictions are color‐grouped as follows: South America (blue), Europe (gray), Africa (green), Australasia (orange/red), Antarctica (purple), all other countries (yellow), and High Seas (black). The proportions of time spent by white‐chinned petrels within the High Seas and in breeding jurisdictions are labeled by white and black text, respectively. (b–d) Fisheries overlap within all Exclusive Economic Zones combined (EEZ, white bar) and in the High Seas area of competency for each Regional Fisheries Management Organization (RFMO) and CCAMLR (Commission for the Conservation of Antarctic Marine Living Resources) (gray‐scale bars) is shown for (b) pelagic longline, (c) demersal longline and (d) trawl fisheries. The percentage of overlap in the High Seas (across all RFMOs) is labeled. RFMO: IATTC, Inter‐American Tropical Tuna Commission; ICCAT, International Commission for the Conservation of Atlantic Tunas; IOTC, Indian Ocean Tuna Commission; SEAFO, South East Atlantic Fisheries Organization; SIOFA, Southern Indian Ocean Fisheries Agreement; SPRFMO, South Pacific Regional Fisheries Management Organization; WCPFC, Western and Central Pacific Fisheries Commission.

White‐chinned petrels spent a substantial proportion of time in the High Seas, although this varied by metapopulation, from 54.6% to 71.4% for Indian Ocean populations to 18.4% to 33.8% for Atlantic populations (Figure 6a; Appendix S3: Table S4). Most overlap with trawl and demersal longline fisheries was within EEZs (85.6%–100%), except for white‐chinned petrels from the Falklands and South Georgia, which had substantial overlap with demersal longliners in the High Seas (37.0% and 48.2%, respectively), specifically South Korean vessels operating outside of the competency of any RFMO (Figures 4m–o and 6c–d). Pelagic longline overlap in the High Seas was substantial for Atlantic and Pacific populations (53.3%–80.1%) and comprised between a third and half of the overlap for Indian Ocean populations (27.2%–46.3%; Figure 6b). Fisheries overlap in the High Seas was in areas under the jurisdiction of different RFMOs: IATTC for white‐chinned petrel populations from the Pacific, IOTC for birds from the Prince Edward Islands, and ICCAT for Crozet, Kerguelen, and Atlantic populations (Figure 6b; Appendix S3: Table S4).

**FIGURE 7:**
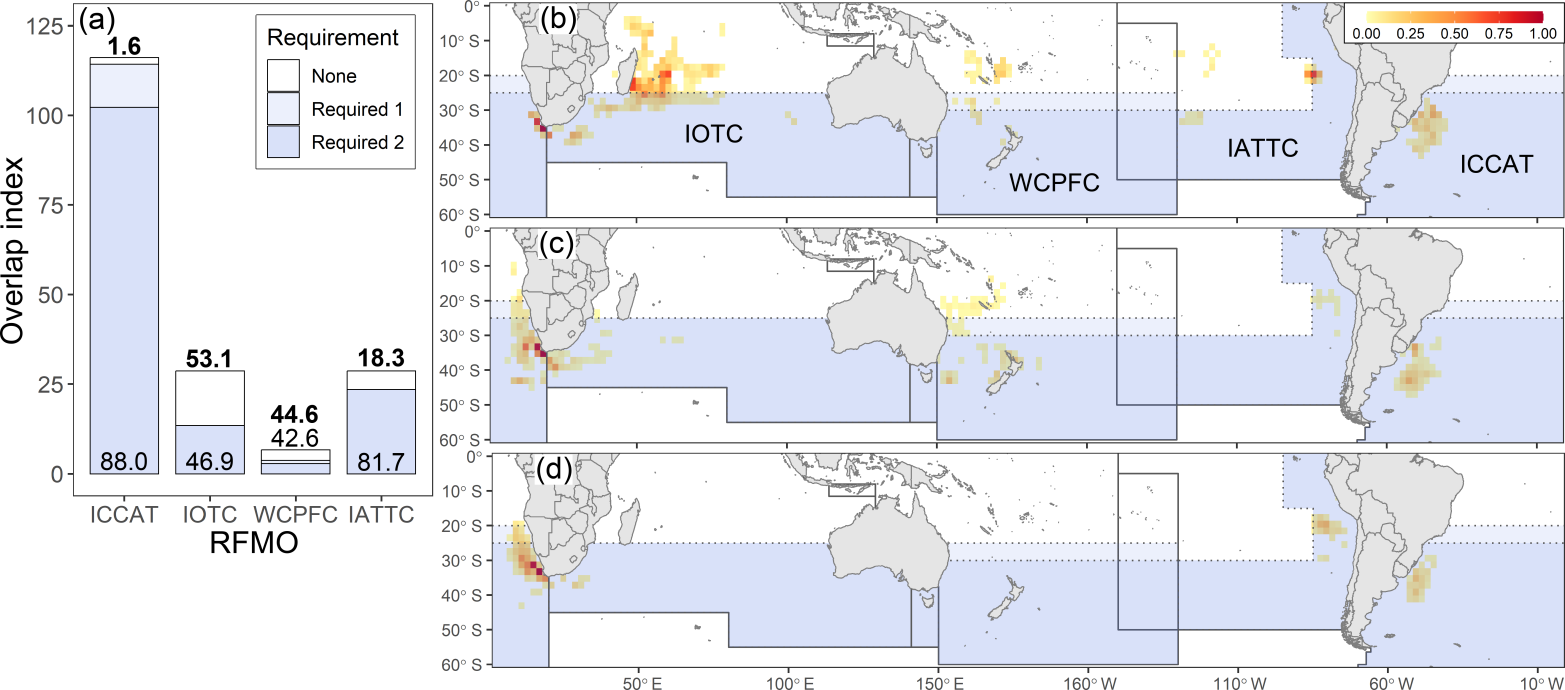
Species‐level overlap of white‐chinned petrels (*Procellaria aequinoctialis*) with pelagic longline fisheries within each Regional Fisheries Management Organization (RFMO) according to seabird bycatch mitigation requirements. Mitigation requirements are geographically defined within each RFMO and were summarized as follows: (1) no mitigation measures required (white), (2) one measure required (lightest blue), and (3) two measures required (light blue). (a) Fisheries overlap summed across the year is shown within each RFMO, with the percentage of overlap in regions without requirements and in regions with two measures indicated by the upper number in bold and the lower number, respectively. Values for each RFMO represent fisheries overlap within EEZs and the High Seas combined (unlike Figure 6). (b–d) Fisheries overlap in relation to the geographical boundaries of seabird bycatch mitigation requirements during (b) prelaying (October–November), (c) breeding (December–April), and (d) nonbreeding (May–September). The boundaries of each RFMO are shown by solid gray lines, and within each RFMO, changes to bycatch mitigation regulations are shown by dotted gray lines. Fisheries overlap was calculated for each population, weighted by population size, summed across gear types, and scaled to a maximum index value of 1. See Appendix S2: Section S6 for details on mitigation measures.

At the species level, most overlap (78.8%) of white‐chinned petrels with pelagic longline fisheries occurred in regions where fishing vessels are required by RFMOs to use seabird bycatch mitigation measures (either hook‐shielding devices or at least two of weighted branch lines, night setting, and tori lines). However, the percentage of overlap within regions with mitigation requirements varied among RFMOs, from 42.6% within the WCPFC to 88.0% within the ICCAT area of competency (Figure 7a). Similarly, while 14% of total petrel‐longline overlap occurred within regions with no requirement to use bycatch mitigation, there was large variation among RFMOs, accounting for up to around half of fisheries overlap within IOTC (53.1%) and WCPFC (44.6%) areas of competency. Notable hotspots of white‐chinned petrel overlap with fisheries in regions with no mitigation requirements were in the southeast Pacific Ocean (~20° S, 95° W; IATTC) and in the southwest Indian Ocean (~20–25° S, 50° E; IOTC) (Figure 7b–d).

## DISCUSSION

We combined seabird tracking and fisheries datasets to map the year‐round distributions and fisheries overlap of white‐chinned petrels. Migratory connectivity analyses showed that populations were more clustered within than among genetically distinct populations. Fisheries overlap varied considerably: lowest for Pacific and highest for Indian Ocean populations, depending on the gear type. Overlap was elevated during the nonbreeding period from all gear types for Indian Ocean populations and from demersal longline and trawl fisheries for birds from South Georgia. The flag states contributing most to fisheries overlap scores were the High Seas longline fleets of Japan, Taiwan, South Korea, and Spain, and the longline and trawl fleets of South Africa, Namibia, and Argentina. Our results indicate that spatial structuring and migratory connectivity of white‐chinned petrel populations shape their exposure to threats such as bycatch, which has implications for conservation.

### Year‐round distributions and migratory connectivity

Previous tracking studies have described the at‐sea distributions, foraging behavior, and overlap with fisheries of white‐chinned petrels from Indian Ocean and South Georgia populations (e.g., Clay et al., 2019b; Péron et al., 2010; Rollinson et al., 2018; Weimerskirch et al., 1999), whereas little was known about birds breeding at other islands. Our study showed that populations from adjacent island groups generally used different areas during prelaying and breeding but mixed to some degree during the nonbreeding season. Nonbreeding white‐chinned petrels tended to concentrate in smaller core areas, presumably because birds are freed from central‐place foraging constraints and fly less (Mackley et al., 2011), and because highly productive waters in nonbreeding areas would support higher concentrations of birds. Indeed, our weighted maps highlight elevated white‐chinned petrel densities around the Patagonian Shelf, driven by the very large South Georgia population, as well as high densities in the Humboldt and Benguela upwelling regions. Using spatial scales appropriate for geolocator data resulted in densities smoothed over large areas, and we acknowledge that aggregations at finer scales may be orders of magnitude higher than the greatest densities indicated here (>5000 individuals 100 km^−2^).

While several lines of evidence have indicated there are two white‐chinned petrel sub‐species, *P. a. steadi* in the Pacific Ocean and *P. a. aequinoctialis* in the Atlantic and Indian Oceans (Techow et al., 2009, 2016), recent genomic evidence suggested a three‐region population structure according to ocean basin, with some structure within metapopulations (Rexer‐Huber et al., 2019). The combined year‐round distributions generally match this ocean‐basin structure, with greater connectivity (less mixing) among than within ocean basins, and are broadly consistent with the idea that divergent nonbreeding distributions could be an important driver of genetic differentiation (Friesen et al., 2007; Rayner et al., 2011). Indeed, the moderate overlap of populations wintering off southern Africa suggests there may be genetic mixing among Indian Ocean populations.

### Overlap with fisheries

There was substantially greater variation in fisheries overlap among than within metapopulations at the scale of ocean basins, and patterns of overlap based on the two sources of fishing effort were broadly similar. Logbook and AIS data are complementary sources of information, but we acknowledge that a major limitation of our analyses was the inability to determine whether differences in fisheries‐overlap scores were due to the data source or time period. Despite this, the geographic distributions of fishing effort were similar, and effort values for pelagic longline fisheries were highly correlated. The greater discrepancy between data sources for demersal longline may reflect a combination of changes in effort (e.g., reductions in Argentinian effort from 2001 to 2010; Favero et al., 2013), the recent appearance of distant‐water fleets (e.g., South Korea) in the AIS data, as well as the potential presence of IUU fishing in the Indian Ocean (Weimerskirch et al., 2020) and within the Argentine EEZ (see below; Agnew et al., 2009; Welch et al., 2022). We also acknowledge that our study was limited by the spatiotemporal scale of biologging and fisheries datasets, and given that overlap indices are highly scale‐dependent, large‐scale overlap may not reflect finer‐scale interactions (Carneiro et al., 2022; Corbeau et al., 2021). A range of environmental, behavioral, and operational factors also complicate the links between large‐scale overlap, interaction, and mortality (Jiménez et al., 2012, 2016; Orben et al., 2021), and mortality risk can be reduced substantially through the use of best‐practice mitigation (e.g., Da Rocha et al., 2021; Jiménez et al., 2020). Nonetheless, at large scales, bycatch hotspots generally occur where densities of seabirds and fishing effort are highest (Jiménez et al., 2020; Yeh et al., 2013). For example, our analysis of white‐chinned petrel captures by New Zealand fleets shows a good correspondence with fisheries‐overlap scores (Appendix S4).

Fisheries‐overlap hotspots generally corresponded with regions and fisheries for which white‐chinned petrel bycatch has been reported since the 1990s (Appendix S1: Table S1). Overlap of white‐chinned petrels with demersal longline and trawl fisheries was particularly high on the Patagonian Shelf and surrounding areas, supporting previous work documenting high risk for the South Georgia population (Clay et al., 2019b; Frankish et al., 2020; Phillips et al., 2006). Indeed, it was estimated that >10,000 white‐chinned petrels were killed per year by fisheries off South America in the early 2000s (Phillips et al., 2006). Overlap scores were high for Indian Ocean populations with all three gear types, and hotspots were located off southern Africa and, to a lesser extent, around breeding colonies. Mortality of white‐chinned petrels in South African and Namibian longline and trawl fisheries was historically high, with >20,000 birds killed per year until bird‐scaring lines were introduced in the mid‐late 2000s (Da Rocha et al., 2021; Maree et al., 2014; Paterson et al., 2019; Petersen, Honig, Ryan, & Underhill, 2009; Petersen, Honig, Ryan, Underhill, & Goren, 2009; Watkins et al., 2008). Similarly, >10,000 birds were killed per year by demersal longliners around breeding colonies in the southwest Indian Ocean in the late 1990s and early 2000s, until effective fisheries management combined with reduced fishing effort reduced bycatch by several orders of magnitude (Delord et al., 2005, 2010). Fisheries‐overlap scores for Pacific populations were generally low, which reflects the relatively modest mortality of birds in New Zealand domestic fisheries (c. 1600 white‐chinned petrels per year, mostly killed by trawlers; Edwards et al., 2023). Data on bycatch rates are limited for distant‐water longline fleets because of the generally low observer coverage (commonly <5%; Jiménez et al., 2020), but for some populations of white‐chinned petrels, more than half of the overlap was with pelagic longliners in the High Seas. Enforcement of best‐practice seabird‐bycatch mitigation and monitoring of seabird bycatch remains a major challenge in the High Seas for flag states and RFMOs (Jiménez et al., 2020; Phillips, 2013).

### Implications for management

The white‐chinned petrel is not only the commonest bycaught seabird species in the Southern Ocean, but because it is a relatively deep diver and returns hooks to the surface, it also facilitates the capture of albatrosses (Jiménez et al., 2012). We have identified regions and fishing fleets for which effective fisheries management can benefit not just multiple populations of white‐chinned petrels, but also other seabirds that may be even more threatened by bycatch (Phillips et al., 2016). Over the past decades, mandatory bycatch‐mitigation regulations have led to substantial reductions in seabird mortality in some regions (Phillips et al., 2016). For example, Indian Ocean white‐chinned petrel populations have mostly stabilized since the enforcement of tougher regulations in demersal longline fisheries (Dasnon et al., 2022; Dilley et al., 2017); the discrepancy between the high overlap with pelagic longline fisheries and the favorable status of Indian Ocean populations suggests that it was the demersal fleets off southern Africa that posed the greatest risk.

Despite recent progress, bycatch mitigation measures in most RFMOs fall short of best practices recommended by the Agreement for the Conservation of Albatrosses and Petrels (ACAP) (Baker et al., 2024). For example, while ACAP recommends pelagic longline vessels employ all three of the following measures—night setting, branch line weighting, and tori lines—or use hook shielding devices (ACAP, 2024), only two of the three (or hook shielding devices) are mandated by all tuna RFMOs (see Appendix S2: Section S6). The spatial coverage of these regulations varies by RFMO: two of the three measures are required in less than 50% of hotspots between white‐chinned petrels and fisheries within IOTC and WCPFC convention areas, compared with over 80% within ICCAT and the IATTC. We highlight two fisheries‐overlap hotspots, in the southeast Pacific Ocean (~20° S, 95° W; IATTC) and in the southwest Indian Ocean (~20–25° S, 50° E; IOTC), where there are currently no regulations and where vessels are unlikely to be using mitigation measures. We encourage RFMOs to revisit mitigation requirements and to update them in line with ACAP best practices, particularly in hotspots of overlap between petrels and fisheries. Another concern is the area of substantial overlap with South Korean demersal longliners operating in the High Seas on the edge of the Patagonian Shelf, in line with other recent studies of white‐chinned petrels and wandering albatrosses (*Diomedea exulans*; Carneiro et al., 2022; Frankish et al., 2020). This is particularly concerning because there is no RFMO for demersal fishing in the southwest Atlantic Ocean, and the vessels often disable their AIS, indicating potential IUU activity (Welch et al., 2022). With information so limited, immediate efforts should be taken by flag states to address seabird bycatch in this fishery.

Low observer coverage within most RFMOs is a major barrier to progress and to a proper understanding of bycatch rates and implementation of bycatch mitigation, while there is often no independent monitoring of compliance (Jiménez et al., 2020; Phillips et al., 2016). We highlight regions and fleets where engagement efforts should be focused. Firstly, New Zealand populations overlapped with pelagic longliners from Japan and Spain in the High Seas off Chile and Peru, where fisheries are managed by the IATTC and where white‐chinned petrels are frequently observed around Japanese vessels (Sato et al., 2016), yet little is known about bycatch rates. Secondly, the white‐chinned petrel is the seabird caught most frequently in longline and gillnet fisheries in Peru (Mangel et al., 2011); our study shows these are predominantly from New Zealand populations. Small‐scale fisheries such as these could not be considered in our study, as vessels are typically not fitted with AIS and rarely report to national fisheries agencies or RFMOs. Therefore, fisheries overlap—and by inference, bycatch risk—is underrepresented where small‐scale fisheries operate in Peru, Chile, Brazil, and likely elsewhere (Frankish et al., 2020; Mangel et al., 2011). Overall, we recommend further engagement with fisheries managers, companies, and fishers regulating or operating vessels in the fleets identified here to ensure mandatory implementation and independent monitoring of best‐practice bycatch mitigation, and monitoring of seabird bycatch rates.

## AUTHOR CONTRIBUTIONS

Kalinka Rexer‐Huber, Thomas A. Clay, and Richard A. Phillips conceived and designed the research. Kalinka Rexer‐Huber, Richard A. Phillips, Paulo Catry, Igor Debski, Graham Parker, Peter G. Ryan, Paul M. Sagar, Andrew Stanworth, David R. Thompson, and Henri Weimerskirch collected the tracking data, and Kalinka Rexer‐Huber, Thomas A. Clay, Richard A. Phillips, and Geoffrey N. Tuck collated the fisheries data. Kalinka Rexer‐Huber, Thomas A. Clay, and Raül Ramos processed the data, and Kalinka Rexer‐Huber and Thomas A. Clay performed the analyses. Kalinka Rexer‐Huber, Thomas A. Clay, and Richard A. Phillips wrote the paper, with contributions from all other authors.

## CONFLICT OF INTEREST STATEMENT

The authors declare no conflicts of interest.

## Supporting information


Appendix S1:

Appendix S2:

Appendix S3:

Appendix S4:

## Data Availability

Processed geolocator data can be downloaded from the BirdLife International Seabird Tracking Database (https://data.seabirdtracking.org/; dataset ids: 439, 635, 1500, 1558, 1582, 1607, 1606, 2024, 2029, 2030, 2032). These datasets and the R code to process geolocator data are available on Zenodo in Clay (2025) at https://doi.org/10.5281/zenodo.14827306. Fishing effort data are available from Global Fishing Watch at a 0.01° resolution in “Version 2.0: Fishing effort data for 2012‐2020” at https://globalfishingwatch.org/data‐download/datasets/public‐fishing‐effort. Logbook effort data are available from the Inter‐American Tropical Tuna Commission (IATTC) at https://www.iattc.org/en-US/Data/Public-domain (dataset title: Tuna and billfish EPO longline catch and effort aggregated by year, month, flag, 5° × 5°; dataset file name: PublicLLTunaBillfish.zip), the International Commission for the Conservation of Atlantic Tunas (ICCAT) at https://www.iccat.int/en/accesingdb.html (sampling fishing statistics and fish sizes; spatio‐temporal estimates of overall Atlantic Fishing Effort for Longline fleets; file name: Effdis LL, accessible at https://www.iccat.int/Data/EFFDIS_LL2000-2023.csv), the Indian Ocean Tuna Commission (IOTC) at https://iotc.org/data/datasets/latest/CE/Longline (Reference ID: IOTC‐DATASETS‐LATEST‐CE‐LONGLINE), and the Western & Central Pacific Fisheries Commission (WCPFC) at https://www.wcpfc.int/folder/public-domain-data (dataset file name: LONGLINE by year, month, flag). Demersal longline data for Argentina, Chile, Namibia, and South Africa (Tuck et al., 2015) are available in Dryad at https://doi.org/10.5061/dryad.7f63m. Trawl data for Uruguay and Namibia (Clay et al., 2019a) are available in Dryad at https://doi.org/10.5061/dryad.k540b54. Monthly demersal longline and trawl fishing effort data aggregated by 5° grid for each fishing fleet and month and year(s) are commercially sensitive and not available publicly; these data are available to qualified researchers as follows: (1) Commission for the Conservation of Antarctic Marine Living Resources (CCAMLR) data for 2000–2020: Daphnis De Pooter (daphnis.depooter@ccamlr.org; Science Data Officer, CCAMLR); (2) Falkland Islands Government Fisheries Department data for Falkland Islands, 1990–2009: Alexander Arkhipkin (aarkhipkin@naturalresources.gov.fk; Senior Fisheries Scientist, Falkland Islands Government Fisheries Department); and (3) Ministry for Primary Industries (MPI) data for New Zealand, 1990–2009: Peta Abernethy (peta.abernethy@mpi.govt.nz; Data Analyst, Fisheries Science and Information, Fisheries New Zealand, MPI). Requests for Australian Fisheries Management Agency (AFMA) data for Australia (1990‐2012) should be submitted via the AFMA Data Request Form (available at https://www.afma.gov.au/reporting-and-accountability/fisheries-management-policies/information-disclosure-fmp-12) and emailed to data.request@afma.gov.au.
