# Supplementary material for: Metapopulation distribution shapes year‐round overlap with fisheries for a circumpolar seabird
Source: Ecol Appl. 2025 Apr 21;35(3):e70019. doi: 10.1002/eap.70019 (PMC12010314; doi:10.1002/eap.70019)
Supplement: Supplementary file 1 — Appendix S1: [file EAP-35-e70019-s004.pdf]

Supporting Information. Kalinka Rexer-Huber, Thomas A. Clay, Paulo Catry, Igor Debski, Graham Parker, Raül Ramos, Bruce C. Robertson, Peter G. Ryan, Paul M. Sagar, Andrew Stanworth, David R. Thompson, Geoffrey N. Tuck, Henri Weimerskirch, Richard A. Phillips. 2025. Metapopulation distribution shapes year-round overlap with fisheries for a circumpolar seabird. Ecological Applications.

Appendix S1: Review of white-chinned petrel bycatch in global fisheries

Table S1. Review of white-chinned petrel (WCP) (*Procellaria aequinoctialis*) bycatch in global fisheries, grouped by ocean basin. Values are the estimated number of birds bycaught in each fishery per year, and were reported directly in the references cited or were estimated (grey shaded cells) by 1) dividing the total number of bycaught WCP by the number of fishing seasons, 2) scaling up a WCP bycatch rate by the total effort or 3) multiplying the total or estimated seabird bycatch by the proportion of bycatch represented by WCP. For some flag states (e.g., Japan), values are only provided for observed effort as the total effort was not reported. DLL = demersal longline, PLL = pelagic longline, All = demersal and pelagic longline and trawl combined; FAO = Food and Agriculture Organization of the United Nations; All years = bycatch reported for all years of study; Year = bycatch reported just for one year; Per year = mean bycatch reported across years of study; SL = streamer lines, NS = night setting, LW = line weighting; Est. = estimated; CCAMLR = Convention for the Conservation of Antarctic Marine Living Resources.

Ocean basin	Fishery or flag state (FAO code)	Gear type	Years	Time scale	No. fishing seasons	No. bycaught		Use of mitigation	Reference	Notes
						Total	Per year			
Indian/ Southern	Crozet, France (FRA)	DLL	2001/02-2002/03	All years	2	1,740	**870**	Variable	Delord et al. (2005)	WCP 95% of total seabird bycatch.
	Kerguelen, France (FRA)	DLL				23,451	**11,726**			WCP 97% of total seabird bycatch.
	Prince Edward, South Africa (ZAF)	DLL	1996/97-1999/00	All years	4	1,400	**350**	Variable	Nel et al. (2002)	WCP 80% of total seabird bycatch.
	Japan (JPN)	PLL	1992-2009	All years	17	147	**9**	None	Inoue et al. (2011a)	Values only for observed effort.
	Spain (ESP)	PLL	2011-2015	All years	5	8	**2**	Variable	Fernández-Costa et al. (2016)	
	Taiwan (TWN)	PLL	2004-2007	All years	4	115	**29**	None	Huang and Liu (2010)	
			2010-2016	All years	6	2,405	**401**	Variable	Huang (2017)	Est. bycatch scaled up assuming 8.2% observer coverage rate (mean of 3.0-10.4% range).
Pacific/ Southern	Chile (CHL)	DLL	2002	Year	1	-	**437**	None	Moreno et al. (2006)	All birds caught were WCP.
	Japan, Australian waters (JPN)	PLL	1989-1995	All years	6	1,495	**249**	Low	Gales et al. (1998)	
	Japan (JPN)	PLL	1992-2010	All years	19	2	**<1**	None	Inoue et al. (2011b)	Values only for observed effort.

	New Zealand (NZL)	PLL	2007-2020	Year		-	**115**	Some	Edwards et al. (2023)	
		DLL	2007-2020	Year		-	**554**	Some	Edwards et al. (2023)	
		Trawl	2007-2020	Year		-	**963**	Some	Edwards et al. (2023)	
	Taiwan (TWN)	PLL	2002-2006	All years	5	228	**46**	None	Huang and Yeh (2011)	
			2010-2016	All years	6	193	**32**	Variable	Huang (2017)	See above.
Atlantic/ Southern	Argentina (ARG)	DLL	1999-2001	Per year	-	-	**233**	None	Favero et al. (2003)	WCP 20% of est. 1,160 seabirds killed annually.
			2001-2010	All years	10	2,180	**218**	None	Favero et al. (2013)	
		Trawl	2006-2007	Year	1	-	**0**	None	Favero et al. 2011	Heavy interactions
	Brazil (BRA)	DLL	1990s	Per year	-	-	**724**	None	Olmos et al. (2000)	
		PLL	1990s	Per year	-	-	**3,084**	None		
		PLL	2001-2007	All years	7	47	**7**	None	Bugoni et al. (2008)	
	Falklands, United Kingdom (GBR)	DLL	2001/2002	Year	1	-	**8**	Moderate (SL)	Reid et al. (2004)	
		Trawl	2002/2003	Year	1	-	**35**	None	Sullivan et al. (2006)	
	Japan (JPN)	PLL	1997-2009	All years	14	47	**4**	None	Inoue et al. (2012)	Values only for observed effort.
	Namibia (NAM)	DLL	2000-2006	Per year	7	-	**15,865**	None	Petersen et al. (2009a)	WCP captures 95% of total seabirds.
			2009-2012	Per year	4	-	**17,610**	Minimal	Paterson et al. (2019))	WCP captures 86% of 20,567 total seabirds in 2010.
			2018	Year	1	-	**180**	Strong (SL, NS)	Da Rocha et al. (2021)	Assumes WCP capture % similar to pre-regulation period.
		Trawl	2009-2010	All years	2	6,610	**3,305**	None	Da Rocha et al. (2021)	WCP 54% of seabird heavy interactions.
			2016-2018	All years	2	891	**490**	Moderate/ Strong (SL)	Da Rocha et al. (2021)	WCP 31% of seabird heavy interactions.
	South Africa including foreign fleets (ZAF)	DLL	1994	Year	1	-	**8,000**	None	Barnes et al. (1997)	WCP captures 100% of total seabirds.
			2000-2006	Per year	7	-	**88**	Minimal	Petersen et al. (2009a)	WCP captures 36% of total seabirds.
		PLL	1998-2005	All years	8	13,185	**1,650**	Variable	Petersen et al. (2009b)	WCP captures 69% of total seabirds.
		Trawl	2004-2005	Year	1	-	**1,500**	None	Watkins et al. (2008)	WCP captures 10% of total seabirds.
			2006-2010	All years	4	124	**31**	Strong (SL)	Maree et al. (2014)	Heavy interactions and mortalities.
	Taiwan (TWN)	PLL	2010-2016	All years	6	2,878	**480**	Variable	Huang (2017)	See above.

	South Georgia, United Kingdom (GBR)	DLL	1996-1997	All years	2	5,595	**2,798**	Minimal	Collins et al. (2021)	
			2000-2019	All years	20	294	**15**	Strong (LW, NS, SL)		
	Uruguay (URY)	PLL	2004-2007	All years	4	239	**60**	None	Jiménez et al. (2010)	
All regions	IUU fishing	DLL	1996-2003	All years	8	116,130	**14,516**	None	CCAMLR (2003)	Est. within the CCAMLR Convention Area.

References

Barnes, K.N., Ryan, P.G., Boix-Hinzen, C., 1997. The impact of the hake *Merluccius* spp. longline fishery off South Africa on procellariiform seabirds. *Biological Conservation* 82:227–234.

Bugoni, L., Mancini, P.L., Monteiro, D.S., Nascimento, L., Neves, T.S., 2008. Seabird bycatch in the Brazilian pelagic longline fishery and a review of capture rates in the southwestern Atlantic Ocean. *Endangered Species Research* 5:137–147.

CCAMLR. 2003. *Report of the 22nd Meeting of the Scientific Committee*. Hobart, Australia, CCAMLR.

Collins, M.A., Hollyman, P.R., Clark, J., Soeffker, M., Yates, O., Phillips, R.A., 2021. Mitigating the impact of longline fisheries on seabirds: Lessons learned from the South Georgia Patagonian toothfish fishery (CCAMLR Subarea 48.3). *Marine Policy* 131:104618.

Da Rocha, N., Oppel, S., Prince, S., Matjila, S., Shaanika, T.M., Naomab, C., Yates, O., Paterson, J.R.B., Shimooshili, K., Frans, E., Kashava, S., Crawford, R., 2021. Reduction in seabird mortality in Namibian fisheries following the introduction of bycatch regulation. *Biological Conservation* 253:108915.

Delord, K., Gasco, N., Weimerskirch, H., Barbraud, C., 2005. Seabird mortality in the Patagonian toothfish longline fishery around Crozet and Kerguelen Islands, 2001-2003. *CCAMLR Science* 12:53–80.

Edwards, C.T.T., Peatman, T., Goad, D., Webber, D.N., 2023. *Update to the risk assessment for New Zealand seabirds. New Zealand Aquatic Environment and Biodiversity Report 314*. Wellington, New Zealand, Ministry of Fisheries.

Favero, M., Blanco, G., Copello, S., Seco Pon, J.P., Patterlini, C., Mariano-Jelicich, R., Garcia, G., Belon, M.P., 2013. Seabird bycatch in the Argentinean demersal longline fishery, 2001-2010. *Endangered Species Research* 19:187–199.

Favero, M., Blanco, G., García, G., Copello, S., Seco Pon, J.P., Frere, E., Quintana, F., Yorio, P., Rabuffetti, F., Cañete, G., Gandini, P., 2011. Seabird mortality associated with ice trawlers in the Patagonian shelf: effect of discards on the occurrence of interactions with fishing gear. *Animal Conservation* 14:131–139.

Favero, M., Khatchikian, C.E., Arias, A., Rodriguez, P.S., Canete, G., Mariano-Jelicich, R., 2003. Estimates of seabird by-catch along the Patagonian Shelf by Argentine longline fishing vessels, 1999–2001. *Bird Conservation International* 13:273–281.

Fernández-Costa, J., Ramos-Cartelle, A., Carroceda, A., Mejuto, J., 2016. *Interaction between seabirds and spanish surface longline targeting swordfish in the Indian Ocean (lat ≥ 25°) during the period 2011-2015*. Victoria, Seychelles, Indian Ocean Tuna Commission.

Gales, R., Brothers, N., Reid, T., 1998. Seabird mortality in the Japanese tuna longline fishery around Australia, 1988-1995. *Biological Conservation* 86:37–56.

Huang, H.-W., 2017. *Distribution of seabirds bycatch of Taiwanese longline fleets in Southern Ocean between 2010 and 2016*. Wellington, New Zealand, Commission for the Conservation of Southern Bluefin Tuna.

Huang, H.-W., Liu, K.-M., 2010. Bycatch and discards by Taiwanese large-scale tuna longline fleets in the Indian Ocean. *Fisheries Research* 106:261–270.

Huang, H.-W., Yeh, Y.-M., 2011. Impact of Taiwanese distant water longline fisheries on the Pacific seabirds: finding hotspots on the high seas. *Animal Conservation* 14:562–574.

Inoue, Y., Yokawa, K., Minami, H., Ochi, D., 2011a. *Preliminary view of bycatch hotspot: bycatch distribution in the IOTC area of the southern hemisphere*. Maldives, Indian Ocean Tuna Commission.

Inoue, Y., Yokawa, K., Minami, H., Ochi, D., Sato, N., Katsumata, N., 2011b. *Distribution of seabird bycatch at WCPFC and the neighboring area of the southern hemisphere (WCPFC-SC7-2011/EB-WP-07).* Pohnpei, Federated States of Micronesia, Western Central Pacific Fisheries Commission.

Inoue, Y., Yokawa, K., Minami, H., Ochi, D., Sato, N., Katsumata, N., 2012. Distribution of seabird by-catch using data collected by Japanese observers in 1997-2009 in the ICCAT area. *Collective Volume of Scientific Papers ICCAT* 68:1738–1753.

Jiménez, S., Abreu, M., Pons, M., Ortiz, M., Domingo, A., 2010. Assessing the impact of the pelagic longline fishery on albatrosses and petrels in the southwest Atlantic. *Aquatic Living Resources* 23:49–64.

Maree, B.A., Wanless, R.M., Fairweather, T.P., Sullivan, B.J., Yates, O., 2014. Significant reductions in mortality of threatened seabirds in a South African trawl fishery. *Animal Conservation* 17:520–529.

Moreno, C.A., Arata, J.A., Rubilar, P., Hucke-Gaete, R., Robertson, G., 2006. Artisanal longline fisheries in Southern Chile: Lessons to be learned to avoid incidental seabird mortality. *Biological Conservation* 127:27–36.

Nel, D.C., Ryan, P.G., Watkins, B.P., 2002. Seabird mortality in the Patagonian toothfish longline fishery around the Prince Edward Islands, 1996–2000. *Antarctic Science* 14:151–161.

Olmos, F., Bastos, G.C.C., Neves, T., 2000. Estimating seabird bycatch in Brazil. *Marine Ornithology* 28:141.

Paterson, J.R.B., Yates, O., Holtzhausen, H., Reid, T., Shimooshili, K., Yates, S., Sullivan, B.J., Wanless, R.M., 2019. Seabird mortality in the Namibian demersal longline fishery and recommendations for best practice mitigation measures. *Oryx* 53:300–309.

Petersen, S.L., Honig, M.B., Ryan, P.G., Underhill, L.G., Goren, M., 2009a. Seabird bycatch in the demersal longline fishery off southern Africa. *African Journal of Marine Science* 31:205–214.

Petersen, S.L., Honig, M.B., Ryan, P.G., Underhill, L.G., 2009b. Seabird bycatch in the pelagic longline fishery off southern Africa. *African Journal of Marine Science* 31:191–204.

Reid, T.A., Sullivan, B.J., Pompert, J., Enticott, J.W., Black, A.D., 2004. Seabird mortality associated with Patagonian Toothfish (*Dissostichus eleginoides*) longliners in Falkland Islands waters. *Emu - Austral Ornithology* 104:317–325.

Sullivan, B.J., Reid, T.A., Bugoni, L., 2006. Seabird mortality on factory trawlers in the Falkland Islands and beyond. *Biological Conservation* 131:495–504.

Watkins, B.P, Petersen, S.L., Ryan, P.G., 2008. Interactions between seabirds and deep-water hake trawl gear: an assessment of impacts in South African waters. *Animal Conservation* 11:247–254.
