# Supplementary material for: Metapopulation distribution shapes year‐round overlap with fisheries for a circumpolar seabird
Source: Ecol Appl. 2025 Apr 21;35(3):e70019. doi: 10.1002/eap.70019 (PMC12010314; doi:10.1002/eap.70019)
Supplement: Supplementary file 2 — Appendix S2: [file EAP-35-e70019-s001.pdf]

Supporting Information. Kalinka Rexer-Huber, Thomas A. Clay, Paulo Catry, Igor Debski, Graham Parker, Raül Ramos, Bruce C. Robertson, Peter G. Ryan, Paul M. Sagar, Andrew Stanworth, David R. Thompson, Geoffrey N. Tuck, Henri Weimerskirch. Richard A. Phillips. 2025. Metapopulation distribution shapes year-round overlap with fisheries for a circumpolar seabird. Ecological Applications.

Appendix S2: Supplementary Methods and Results

Section S1: Comparing tracking data from Falkland Islands colonies

White-chinned petrels (*Procellaria aequinoctialis*) in the Falkland Islands were tracked from two different colonies, New Island and Kidney Island. We checked for differences in the distributions of petrels from these colonies by plotting Utilization Distribution (UDs) over the annual cycle and calculated overlap scores (Bhattacharya's Affinity [BA] index) using the *adehabitatHR* package (Calenge 2006). There were no obvious differences in the distributions of petrels from the two colonies (Appendix S2: Fig. S1). Overlap scores revealed substantial overlap of the 50% (pre-laying: 0.49, breeding: 0.72, non-breeding: 0.62) and 70% (pre-laying: 0.65, breeding: 0.86, non-breeding: 0.72) UDs and scores for Falkland Islands colonies were greater than for birds from different island groups (Appendix S2: Table S1).

Section S2: Assessing representativeness of tracking datasets

We used the rep.assess function in the *track2kba* package (Beal *et al.* 2021) to assess whether tracking datasets were representative of the distribution of the wider populations (Lascelles *et al.* 2016). The function iteratively selects subsamples of tracks, averages them into a pooled UD and then calculates the proportion of out-of-sample tracking locations within the resulting area (i.e. inclusion rate). A non-linear regression was fitted to the relationship between sample size and inclusion rate with the maximum asymptotic value considered to be the point at which a dataset is fully representative. A value of 70% of this asymptote was deemed sufficient to identify important sites at the population-level (Lascelles *et al.* 2016). As the representativeness values of all datasets were *c.* 90% or greater (Marion: 94.9%, Crozet: 89.5%, Kerguelen: 92.0%, Auckland: 95.5%, Antipodes: 96.3%, Falklands: 91.7%, South Georgia: 95.9%), we deemed our sample sizes to be broadly representative of the year-round distributions of birds from the tracked populations.

Section S3: Migratory connectivity analyses

We quantified migratory connectivity using Mantel correlations (Ambrosini *et al.* 2009; Cohen *et al.* 2018) based on distance matrices of breeding and non-breeding locations. Breeding locations were specified as an individual's colony of origin. For individuals tracked over multiple years, we randomly chose one year of tracking data. Non-breeding locations were based on the centroids of projected positions during July, when birds are most likely to be resident in non-breeding areas. We moved non-breeding centroids occurring on land to the nearest location off land used the points2nearestcell function in the *rSDM* package (Rodriguez-Sanchez 2023). The function requires a rasterized land layer for which cells on land and in the ocean are given values of NA and 1, respectively. Breeding and non-breeding distance matrices were calculated using the *gdistance* package (van Etten 2017). Specifically, distances between

pairs of individual locations were calculated on a projected grid using the costDistance function, which takes into account land masses and re-routes distances around them. Breeding and non-breeding distance matrices were compared using Mantel's tests in package *ncf* (Bjornstad & Cai 2022). Correlations were conducted on all individuals and then separately for all individuals within each metapopulation (Indian, Pacific and Atlantic Oceans) to look at connectivity at both the species- and ocean-basin level.

Section S4: Obtaining logbook and AIS fishing effort data

Monthly logbook effort reported to Regional Fisheries Management Organizations (RFMOs), CCAMLR or national fisheries agencies. Logbook data were requested at a 5° resolution for 2000–2009. For pelagic longline fisheries, logbook data were obtained from the International Commission for the Conservation of Atlantic Tunas (ICCAT), Indian Ocean Tuna Commission (IOTC), Western and Central Pacific Fisheries Commission (WCPFC) and Inter-American Tropical Tuna Commission (IATTC). Logbook data for demersal longline and trawl fisheries were obtained from the Commission for the Conservation of Antarctic Living Marine Resources (CCAMLR) and from national fisheries agencies of Argentina, Australia, Chile, Falklands, Namibia, New Zealand, South Africa, and Uruguay (see Clay *et al.* 2019 for further details). Only cells containing effort from five or more vessels were provided by the Australian Fisheries Management Agency (AFMA), which reduced Australian effort substantially.

Daily satellite AIS data from Global Fishing Watch (GFW). We used the GFW *v.* 2 dataset (https://globalfishingwatch.org/data-download/datasets/public-fishing-effort-10:v20200316, downloaded on 12/03/2021) at a 0.01° resolution for 2012–2020. GFW combines public vessel registries and machine learning to identify fishing vessels and detect when they are actively fishing (Kroodsma *et al.* 2018). Effort datasets were matched with metadata providing the best assignment of flag state and gear type for each vessel. We considered the following gear types: drifting (hereafter pelagic) longliners, set (demersal) longliners, and trawlers. Initial data visualisation revealed unusually high pelagic longline effort off the Patagonian Shelf, well beyond the usual southernmost extent of this fishery (Tuck *et al.* 2003; Clay *et al.* 2019). As there was no independent verification of gear type used by the two Chinese longliners involved, these data were excluded from overlap analyses (advice D. Kroodsma, pers. comm.).

Section S5: Comparing fishing effort and overlap from logbook and AIS data sources

To investigate concordance between vessel logbook and Automatic Identification System (AIS) datasets, we summed pelagic longline effort within each 5° grid cell, after first resampling AIS data to a 5° and monthly resolution. This was done for the period 2016 to 2018, when we had data from both sources. We used a Spearman rank correlation to associate the total number of hooks from logbooks and the total number of fishing hours from AIS data. The two metrics of pelagic longline effort corresponded extremely well (Spearman rank correlation; $S_{158} = 26.2$, $r = 0.90$, $P < 0.001$), especially for grid cells and months (April–September) with the highest effort (Appendix S2: Fig. S2). Logbook demersal longline and trawl data were not available after 2009 and so could not be compared directly with AIS data, which were only available from 2012 onwards.

Section S6: Examining fisheries overlap in relation to RFMO mitigation regulations

We examined the degree to which pelagic longline fisheries-overlap hotspots were covered by RFMO seabird bycatch mitigation requirements. Fisheries overlap scores were calculated for each population and RFMO, weighted according to the population size and summed across all populations and months to create species-level fisheries-overlap hotspot maps (see Methods). We split RMFO regions according to spatial boundaries of regulations (see Fig. 7b-d), which were summarised into the following three categories: 1) no mitigation measures required, 2) one measure required, and 3) two measures required (see below). Fisheries-overlap hotspot maps were overlaid with shapefiles of the geographic boundaries of bycatch mitigation requirements for each RMFO, and the percentage of total overlap under each mitigation category was calculated for each RFMO.

Seabird bycatch mitigation regulations, as of October 2024, are as follows for each RFMO:

- **ICCAT** (Recommendation 07-07) states that fleets are required to use two of three measures – weighted branch lines, night setting and tori lines – when fishing south of 25°S, or at least bird-scaring lines when fishing between 20°S and 25°S (Bell et al. 2024).
- **IOTC** (Resolution 23/07) (IOTC 2023) states that in the area south of 25°S vessels should use at least two of either weighted branch lines, night setting and tori lines, or hook-shielding devices. There are no requirements north of 25°S.
- **WCPFC** (Conservation and Management Measure [CMM] 2018-03) (WCPFC 2018) states that vessels south of 30°S are required to use at least two of either weighted branch lines, night setting and tori lines, or hook-shielding devices. Vessels operating between 25°S and 30°S are required to use one of either weighted branch lines, tori lines or hook-shielding devices. Between 25°S and 23°N vessels are encouraged (but not required) to employ one or more of the mitigation measures listed above (plus several others).
- **IATTC** (Resolution C-11-02) (IATTC 2011) states that in waters south of 30°S plus the area bounded by the coastline at 2°N, west to 2°N-95°W, east to 15°S-85°W and south to 30°S, to use at least two of the following mitigation measures: 1) side-setting with bird curtains and weighted branch lines, night setting, 2) tori lines, 3) weighted branch lines, 4) blue-dyed bait, 5) deep-setting line shooter, 6) underwater setting chute and 7) management of offal discharge, including at least one of 1-4). There are no requirements outside of the area specified above.

References

Ambrosini, R., Møller, A.P,. Saino, N., 2009. A quantitative measure of migratory connectivity. *Journal of Theoretical Biology* 257: 203–211.

Beal, M., Oppel, S., Handley, J., Pearmain, E.J., Morera-Pujol, V., Carneiro, A.P.B., Davies, T.E., Phillips, R.A., Taylor, P.R., Miller, M.G.R., Franco, A.M.A., Catry, I., Patrício, A.R., Regalla, A., Staniland, I., Boyd, C., Catry, P., Dias, M.P., 2021. track2KBA: An R package for identifying important sites for biodiversity from tracking data. *Methods in Ecology and Evolution* 12: 2372-2378.

Bell, J., A. P. B. Carneiro, A. Bielli, S. Jiménez, S. Oppel, R. A. Phillips, H. M. Wade, O. Yates, S. Griffiths, and S. Reeves. 2024. Effectiveness of Conservation and Management Measures for Reducing Seabird Bycatch on Pelagic Longlines in the South Atlantic. Collect. Vol. Sci. Pap. ICCAT 81:1–33.

Bjornstad, O.N., Cai, J., 2022. ncf: Spatial Covariance Functions Available at: https://cran.r-project.org/web/packages/ncf/index.html [Accessed June 15, 2023].

Calenge C., 2006. The package "adehabitat" for the R software: A tool for the analysis of space and habitat use by animals. *Ecological Modelling 197*:516–519.

Clay, T.A., Small, C., Tuck, G.N., Pardo, D., Carneiro, A.P.B., Wood, A.G., Croxall, J.P., Crossin, G.T., Phillips, R.A., 2019. A comprehensive large-scale assessment of fisheries bycatch risk to threatened seabird populations. *Journal of Applied Ecology* 56:1882–1893.

Cohen, E.B., Hostetler, J.A., Hallworth, M.T., Rushing, C.S., Sillett, T.S., Marra, P.P., 2018. Quantifying the strength of migratory connectivity. *Methods in Ecology and Evolution* 9: 513–524.

IATTC. 2011. Resolution C-11-02: Resolution to mitigate the impact on seabirds of fishing for species covered by the IATTC. Inter-American Tropical Tuna Commission.

IOTC. 2023. Resolution 23/07: On Reducing the Incidental Bycatch of Seabirds in Longline Fisheries. Indian Ocean Tuna Commission.

Lascelles, B.G., Taylor, P.R., Miller, M.G.R., Dias, M.P., Oppel, S., Torres, L., Hedd, A., Le Corre, M., Phillips, R.A., Shaffer, S.A., Weimerskirch, H., Small, C., 2016. Applying global criteria to tracking data to define important areas for marine conservation. *Diversity and Distributions* 22, 422–431.

Kroodsma, D.A., Mayorga, J., Hochberg, T., Miller, N.A., Boerder, K., Ferretti, F., Wilson, A., Bergman, B., White, T.D., Block, B.A., Woods, P., Sullivan, B., Costello, C., Worm, B., 2018. Tracking the global footprint of fisheries. *Science* 359, 904–908.

Rodriguez-Sanchez, F. 2023. rSDM: Species distribution and niche modelling in R Available at: https://github.com/Pakillo/rSDM.

Tuck, G.N., Polacheck, T., Bulman, C.M., 2003. Spatio-temporal trends of longline fishing effort in the Southern Ocean and implications for seabird bycatch. *Biological Conservation* 114:1–27.

van Etten, J., 2017. R Package gdistance: Distances and routes on geographical grids. *Journal of Statistical Software* 76:1–21.

WCPFC. 2018. Conservation and Management Measure 2018-03: Conservation and Management Measure to Mitigate the Impact of Fishing for Highly Migratory Fish Stocks on Seabirds. Western and Central Pacific Fisheries Commission.

Table S1. Pairwise spatial overlap in distributions of white-chinned petrels (*Procellaria aequinoctialis*) tracked with geolocators from different island populations during a) pre-laying (October–November), b) breeding (December–April) and c) non-breeding (May–September). Overlap values on the top right and bottom left indicate overlap of 50% and 70% Utilization Distributions (UDs), respectively. Pairwise overlap within the same ocean-basin grouping is shown by light grey shading.

a) Pre-laying

	PEI	CRO	KER	AKL	ANT	FLK	SOG
PEI		0.21	0.00	0.00	0.00	0.00	0.00
CRO	0.38		0.05	0.00	0.00	0.00	0.00
KER	0.03	0.15		0.00	0.00	0.00	0.00
AKL	0.00	0.00	0.00		0.22	0.00	0.00
ANT	0.00	0.00	0.00	0.38		0.00	0.00
FLK	0.00	0.00	0.00	0.00	0.00		0.32
SOG	0.00	0.00	0.00	0.00	0.00	0.46	

b) Breeding

	PEI	CRO	KER	AKL	ANT	FLK	SOG
PEI		0.02	<0.01	0.00	0.00	0.00	0.00
CRO	0.23		0.01	0.00	0.00	0.00	0.00
KER	0.08	0.06		0.00	0.00	0.00	0.00
AKL	0.00	0.00	0.00		0.19	0.00	0.00
ANT	0.00	0.00	0.00	0.33		0.00	0.00
FLK	0.00	0.00	0.00	0.00	0.00		0.62
SOG	0.00	0.00	0.00	0.00	0.00	0.69	

c) Non-breeding

	PEI	CRO	KER	AKL	ANT	FLK	SOG
PEI		0.14	0.10	0.00	0.00	0.00	0.00
CRO	0.29		0.51	0.00	0.00	0.00	0.00
KER	0.28	0.65		0.00	0.00	0.00	0.00
AKL	0.00	0.00	0.00		0.16	<0.01	0.00
ANT	0.00	0.00	0.00	0.49		0.55	0.06
FLK	0.00	0.00	0.00	0.15	0.55		0.26
SOG	0.00	0.00	0.00	0.01	0.15	0.55	

Island populations: PEI = Prince Edward, CRO = Crozet, KER = Kerguelen, AKL = Auckland, ANT = Antipodes, FLK = Falklands, SOG = South Georgia.

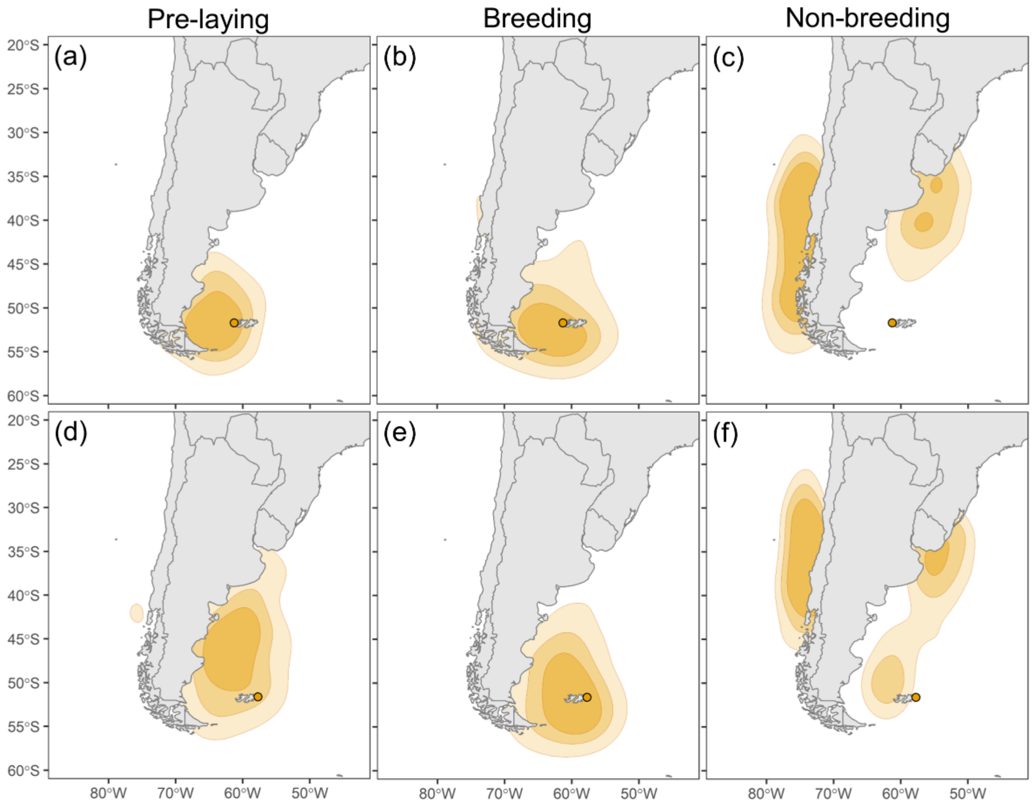

Figure S1. Utilization Distributions (UDs) of white-chinned petrels from New Island (a–c) and Kidney Island (d–f), in the Falkland Islands, during (a, d) pre-laying (October–November), (b, e) breeding (December–April) and (c, f) non-breeding (May–September). UD contours for 30%, 50% and 70% UD are coloured with progressively lighter shades of orange and colony locations are indicated by orange circles.

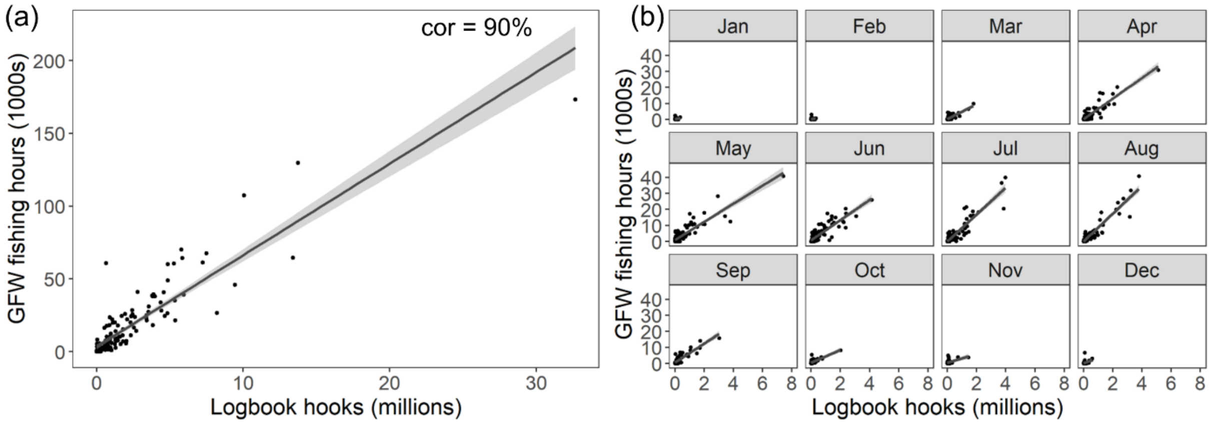

Figure S2. Correlating pelagic longline fishing effort based on vessel logbook data (hooks × 10^6) and Automatic Identification System (AIS) data from Global Fishing Watch (hours × 10^3), a) across the year and b) for each month separately.

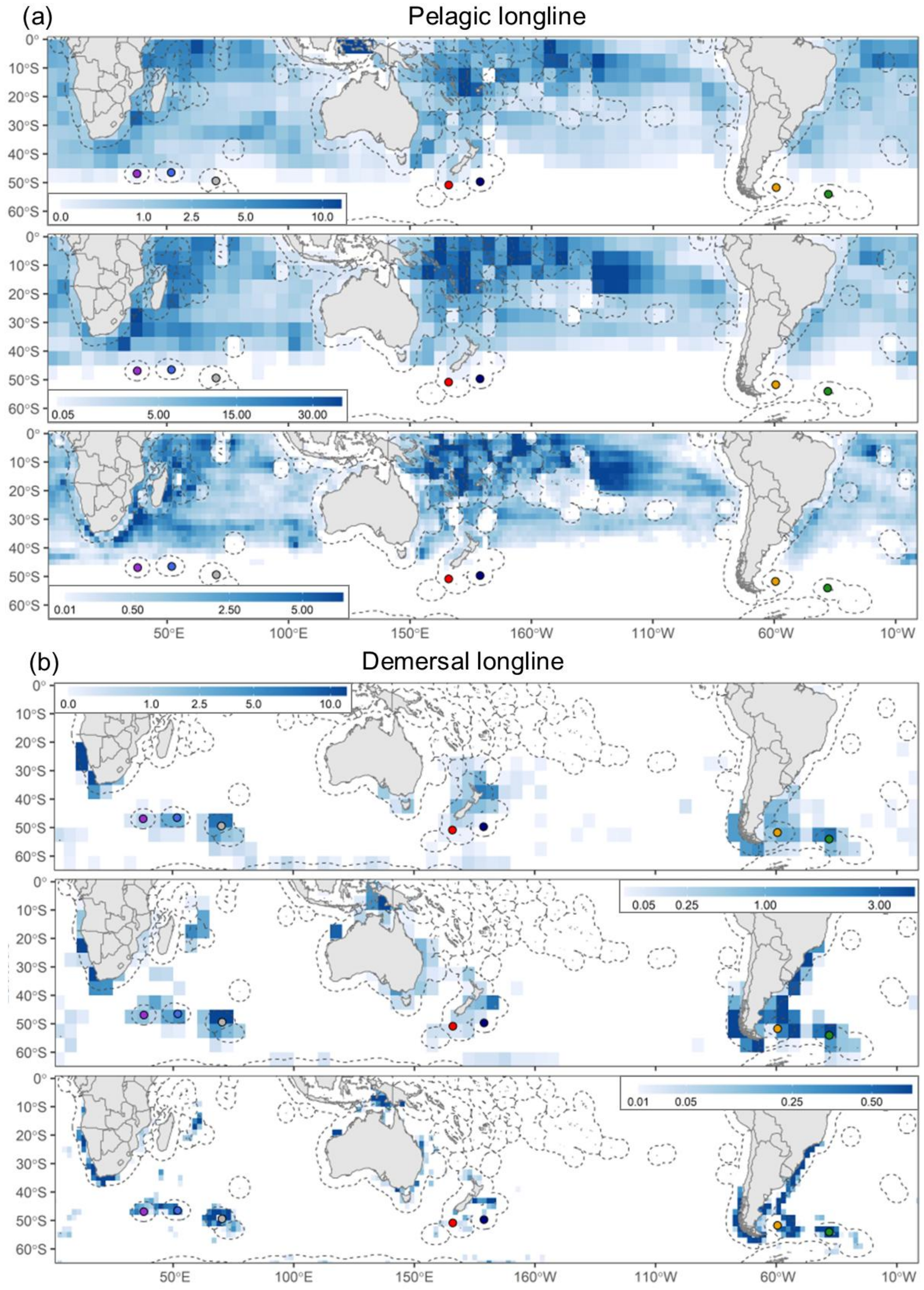
(a)
Pelagic longline
0°
10°S
20°S
30°S
40°S
50°S
60°S
0.0 1.0 2.5 5.0 10.0
0.05 5.00 15.00 30.00
0.01 0.50 2.50 5.00
50°E
100°E
150°E
160°W
110°W
60°W
10°W
(b)
Demersal longline
0.0 1.0 2.5 5.0 10.0
0.05 0.25 1.00 3.00
0.01 0.05 0.25 0.50

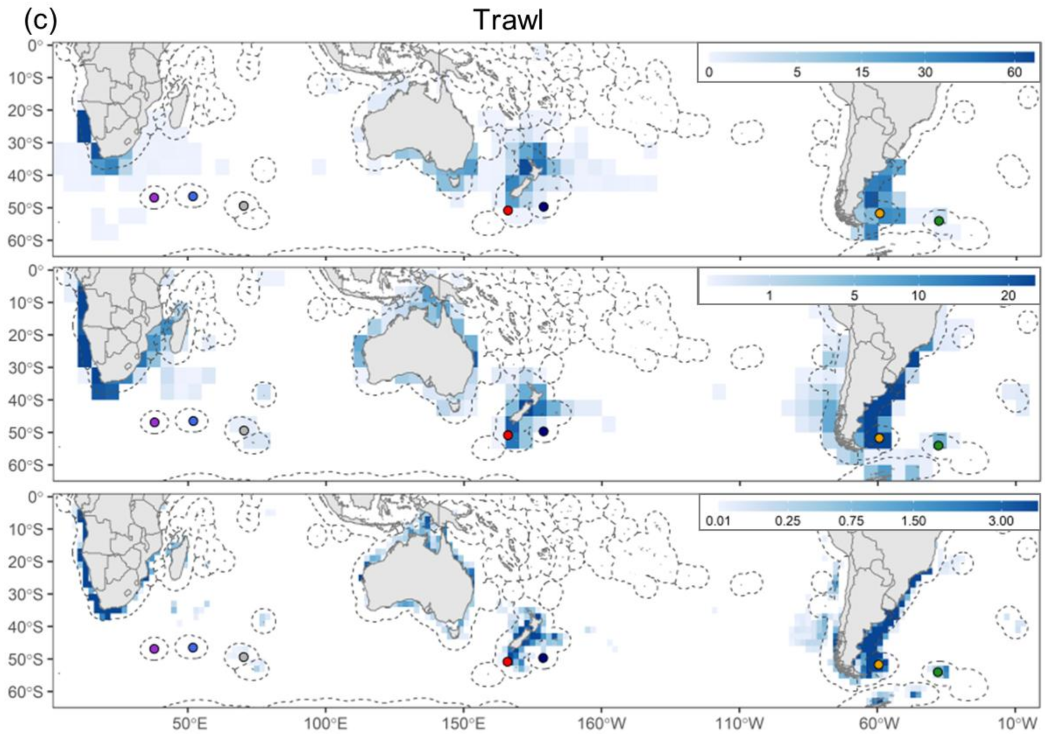

Figure S3. Maps of a) pelagic longline, b) demersal longline and c) trawl fishing effort, according to data source and spatial resolution. For each plot, panels represent from top to bottom, logbook data at a 5° resolution, AIS data at a 5° resolution and AIS data at a 2° resolution. Cells are coloured according to effort score (plotted on a square root scale). To reduce the influence of a few extremely high values, any value greater than the top 1% was set to that reference value. Values were summed across the study period and represent the number of hooks ($\times 10^6$) for logbook longline effort and the number of fishing hours ($\times 10^3$) for logbook trawl and all AIS effort.
