# Supplementary material for: Metapopulation distribution shapes year‐round overlap with fisheries for a circumpolar seabird
Source: Ecol Appl. 2025 Apr 21;35(3):e70019. doi: 10.1002/eap.70019 (PMC12010314; doi:10.1002/eap.70019)
Supplement: Supplementary file 3 — Appendix S3: [file EAP-35-e70019-s002.pdf]

**Supporting Information.** Kalinka Rexer-Huber, Thomas A. Clay, Paulo Catry, Igor Debski, Graham Parker, Raül Ramos, Bruce C. Robertson, Peter G. Ryan, Paul M. Sagar, Andrew Stanworth, David R. Thompson, Geoffrey N. Tuck, Henri Weimerskirch, Richard A. Phillips. 2025. Metapopulation distribution shapes year-round overlap with fisheries for a circumpolar seabird. Ecological Applications.

## Appendix S3: Supplementary fisheries overlap results

**Table S1.** The contribution of each flag state to the summed annual overlap for each white chinned petrel (*Procellaria aequinoctialis*) population (island group) with a) pelagic longline, b) demersal longline and c) trawl fisheries, based on logbook data during 2000–2009. Overlap values are shown divided by 1,000 with the percentage contribution to overall scores in parentheses. Grey-shaded boxes represent flag states that contribute to 5% or more of overlap for that population and gear type.

| a) | ARG | AUS | BRA | CHL | CHN | ESP | FJI | FRA | GBR | JPN | KOR | NAM | NZL | Other | TWN | URY | VUT | ZAF |
|---|---|---|---|---|---|---|---|---|---|---|---|---|---|---|---|---|---|---|
| PEI | – | – | – | – | 8.4 (0.8) | 7.0 (0.7) | – | – | – | 434.5 (43.8) | 54.5 (5.5) | 16.7 (1.7) | – | 7.4 (0.7) | 355.9 (35.9) | – | – | 107.7 (10.9) |
| CRO | – | – | – | – | 2.7 (0.3) | 51.9 (6.6) | – | – | – | 250.1 (31.8) | 13.5 (1.7) | 121.4 (15.5) | – | 5.3 (0.7) | 282.1 (35.9) | – | – | 58.5 (7.4) |
| KER | – | – | – | – | 3.0 (0.4) | 27.7 (3.8) | – | – | – | 168.7 (23.0) | 14.8 (2.0) | 53.1 (7.2) | – | 9.0 (1.2) | 402.2 (54.8) | – | – | 55.3 (7.5) |
| AKL | – | 24.0 (7.1) | – | – | 3.1 (0.9) | 91.9 (27.3) | 5.1 (1.5) | – | – | 161.2 (47.9) | 0.1 (<0.1) | – | 35.1 (10.4) | 3.8 (1.1) | 9.9 (2.9) | – | 2.3 (0.7) | – |
| ANT | – | 0.3 (0.2) | – | – | 4.9 (3.0) | 33.4 (20.5) | 24.5 (15.1) | – | – | 30.9 (19.0) | 3.1 (1.9) | – | 41.9 (25.7) | 8.5 (5.2) | 11.1 (6.8) | – | 4.2 (2.6) | – |
| FLK | – | – | 16.4 (14.8) | – | – | 5.5 (4.9) | – | – | – | 5.4 (4.9) | – | – | – | – | 64.0 (57.6) | 19.8 (17.9) | – | – |
| SOG | – | – | 10.8 (12.6) | – | – | 6.1 (7.2) | – | – | – | 4.3 (5.0) | – | – | – | – | 49.4 (57.8) | 14.8 (17.3) | – | – |

| b) | ARG | AUS | BRA | CHL | CHN | ESP | FJI | FRA | GBR | JPN | KOR | NAM | NZL | Other | TWN | URY | VUT | ZAF |
|---|---|---|---|---|---|---|---|---|---|---|---|---|---|---|---|---|---|---|
| PEI | – | – | – | – | – | – | – | 5.5 (0.3) | – | – | – | 526.0 (28.2) | – | 1.1 (0.1) | – | – | – | 1334.6 (71.5) |
| CRO | – | – | – | – | – | – | – | 121.4 (2.4) | – | 0.1 (<0.1) | – | 4112.4 (81.0) | – | 1.0 (<0.1) | – | – | – | 843.0 (16.6) |
| KER | – | 0.1 (<0.1) | – | 2.9 (0.1) | – | – | – | 648.8 (21.3) | – | 4.4 (0.1) | – | 1941.0 (63.7) | – | 3.1 (0.1) | – | – | – | 446.6 (14.7) |
| AKL | – | 17.9 (6.5) | – | – | – | – | – | - | 0.2 (0.1) | – | – | – | 256.1 (93.4) | – | – | – | – | – |
| ANT | 0.4 (0.1) | – | – | 19.8 (7.1) | – | – | – | – | 1.0 (0.3) | – | – | – | 258.6 (92.5) | – | – | – | – | – |
| FLK | 300.0 (49.5) | – | – | 59.6 (9.8) | – | – | – | – | 246.7 (40.7) | – | – | – | – | – | – | – | – | – |

| | | | | | | | | | | | | | | | | | | |
|---|---|---|---|---|---|---|---|---|---|---|---|---|---|---|---|---|---|---|
| SOG | 282.4<br>(61.9) | – | – | 23.3<br>(5.1) | – | – | – | – | 150.2<br>(33.0) | – | - | – | – | – | – | – | – | – |

| **c)** | **ARG** | **AUS** | **BRA** | **CHL** | **CHN** | **ESP** | **FJI** | **FRA** | **GBR** | **JPN** | **KOR** | **NAM** | **NZL** | **Other** | **TWN** | **URY** | **VUT** | **ZAF** |
|---|---|---|---|---|---|---|---|---|---|---|---|---|---|---|---|---|---|---|
| PEI | – | – | – | – | – | – | – | – | – | – | – | 1745.0<br>(15.6) | – | – | – | – | – | 9437.8<br>(84.4) |
| CRO | – | – | – | – | – | – | – | – | – | – | – | 13642.8<br>(72.7) | – | – | – | – | – | 55123.7<br>(27.3) |
| KER | – | – | – | – | – | – | – | – | – | – | – | 6439.2<br>(65.9) | – | – | – | – | – | 3332.9<br>(34.1) |
| AKL | 8.6<br>(0.2) | 490.8<br>(13.0) | – | – | – | – | – | – | – | – | – | – | 3277.7<br>(86.8) | – | – | – | – | – |
| ANT | 17.3<br>(0.9) | – | – | – | – | – | – | – | – | – | – | – | 1800.3<br>(99.1) | – | – | – | – | – |
| FLK | 8778.2<br>(76.3) | – | – | – | – | – | – | – | 1852.0<br>(16.1) | – | – | – | – | – | – | 882.0<br>(7.7) | – | – |
| SOG | 8160.6<br>(79.9) | – | – | 62.3<br>(0.6) | – | – | – | – | 1421.1<br>(13.9) | – | – | – | – | 0.2<br>(<0.1) | – | 568.0<br>(5.6) | – | – |

Populations: PEI = Prince Edward, CRO = Crozet, KER = Kerguelen, AKL = Auckland, ANT = Antipodes, FLK = Falklands, SOG = South Georgia. Flag states: ARG = Argentina, AUS = Australia, BRA = Brazil, CHL = Chile, CHN = China, ESP = Spain, FJI = Fiji, GBR = United Kingdom of Great Britain and Northern Ireland (including the Falkland Islands), FRA = France JPN = Japan, KOR = South Korea, NAM = Namibia, NZL = New Zealand, Other = any flag state that did not represent at least 2% of overlap for at least one population, TWN = Taiwan, URY = Uruguay, VUT = Vanuatu, ZAF = South Africa. – indicates that there was no fleet for given gear type or no overlap with that flag state.

**Table S2.** The contribution of each flag state to the summed annual overlap for each white chinned petrel population (island group) with a) pelagic longline, b) demersal longline and c) trawl fisheries, based on Automatic Identification System (AIS) data during 2012–2020. Overlap values are shown divided by 1,000 with the percentage contribution to overall scores in parentheses. Grey-shaded boxes represent flag states that contribute to 5% or more of overlap for that population and gear type.

| **a)** | **AUS** | **BRA** | **CHN** | **ESP** | **FJI** | **JPN** | **KOR** | **NAM** | **FRA** | **NZL** | **Other** | **PRT** | **SYC** | **TWN** | **VUT** | **ZAF** |
|---|---|---|---|---|---|---|---|---|---|---|---|---|---|---|---|---|
| PEI | – | – | 9.4 (0.3) | 53.1 (1.7) | – | 457.5 (14.2) | 97.3 (3.0) | 21.9 (0.7) | – | – | 15.0 (0.5) | 19.2 (0.6) | 234.0 (7.3) | 946.1 (29.5) | 10.7 (0.3) | 1346.6 (41.9) |
| CRO | <0.1 (<0.1) | – | 61.6 (1.8) | 301.8 (8.8) | – | 580.6 (16.9) | 78.3 (2.3) | 179.9 (5.2) | – | – | 70.0 (2.0) | 100.4 (2.9) | 23.9 (0.7) | 660.9 (19.2) | 118.9 (3.5) | 1267.6 (36.8) |
| KER | – | – | 87.2 (2.8) | 272.1 (8.8) | – | 271.8 (8.8) | 86.6 (2.8) | 89.8 (2.9) | – | – | 135.0 (4.4) | 52.7 (1.7) | 32.7 (1.1) | 964.2 (31.1) | 118.2 (3.8) | 986.3 (31.9) |
| AKL | 37.2 (2.5) | – | 101.2 (12.1) | 790.8 (61.1) | 19.2 (1.5) | 190.8 (14.7) | 3.1 (0.2) | – | 37.8 (2.4) | 55.4 (4.3) | 62.5 (4.8) | 8.6 (0.7) | – | 17.6 (1.4) | 12.4 (1.0) | – |
| ANT | 1.4 (0.2) | – | 178.1 (28.8) | 156.3 (25.3) | 58.5 (9.5) | 63.2 (10.2) | 15.1 (2.4) | – | 6.7 (0.8) | 45.2 (7.3) | 19.1 (3.1) | 0.8 (0.1) | – | 47.6 (7.7) | 33.0 (5.3) | – |
| FLK | – | 54.4 (17.6) | – | 07.4 (13.0) | – | 72.3 (23.3) | – | – | – | – | 1.1 (0.4) | 66.5 (18.3) | – | 85.0 (27.4) | – | – |
| SOG | – | 50.0 (18.4) | – | 48.0 (17.7) | – | 28.0 (10.3) | – | – | – | – | 1.1 (0.4) | 78.6 (29.0) | – | 65.3 (24.1) | 0.3 (0.1) | – |

| **b)** | **AGO** | **ARG** | **AUS** | **BRA** | **CHL** | **FRA** | **GBR** | **KOR** | **NAM** | **NZL** | **Other** | **PAN** | **UKR** | **URY** | **ZAF** |
|---|---|---|---|---|---|---|---|---|---|---|---|---|---|---|---|
| PEI | 0.3 (<0.1) | – | – | – | – | 1.5 (0.2) | – | 26.5 (3.3) | 23.0 (2.8) | – | 7.2 (0.9) | 6.0 (0.7) | – | – | 744.0 (92.0) |
| CRO | 23.0 (2.4) | – | 0.9 (0.1) | – | – | 68.1 (7.2) | – | 118.2 (12.5) | 223.5 (23.7) | – | 9.4 (1.0) | 57.4 (6.1) | – | – | 442.0 (46.9) |
| KER | – | – | 3.8 (0.5) | – | – | 359.5 (46.6) | – | 32.7 (4.2) | 66.9 (8.7) | – | 4.7 (0.6) | 16.6 (2.2) | – | – | 287.7 (37.3) |
| AKL | – | 3.4 (2.4) | 18.9 (13.2) | – | 48.2 (33.8) | – | – | – | – | 70.0 (49.1) | 1.1 (0.8) | – | 0.7 (0.6) | – | – |
| ANT | – | 3.1 (1.2) | 0.2 (0.1) | - | 178.0 (70.6) | – | – | 0.1 (0.1) | – | 68.2 (27.0) | 0.7 (0.3) | – | 1.9 (0.7) | – | – |
| FLK | – | 394.6 (23.8) | - | 9.0 (0.5) | 293.7 (17.7) | – | 91.8 (5.5) | 695.1 (42.0) | – | 0.1 (<0.1) | 1.7 (0.1) | – | 44.1 (2.7) | 92.8 (5.6) | – |
| SOG | – | 230.8 (14.7) | - | 93.2 (5.9) | 216.8 (13.8) | – | 68.1 (4.3) | 827.0 (52.6) | – | 0.1 (<0.1) | 2.7 (0.2) | – | 46.3 (2.9) | 73.0 (4.6) | – |

| **c)** | **AGO** | **ARG** | **AUS** | **CHL** | **CHN** | **ESP** | **GBR** | **KOR** | **NAM** | **NZL** | **Other** | **UKR** | **URY** | **VCT** | **VUT** | **ZAF** |
|---|---|---|---|---|---|---|---|---|---|---|---|---|---|---|---|---|
| PEI | 12.0 (0.2) | – | 0.1 (<0.1) | – | 2.9 (<0.1) | 28.3 (0.4) | 0.3 (<0.1) | 2.1 (<0.1) | 418.5 (5.6) | 0.1 (<0.1) | 51.1 (0.7) | 0.1 (<0.1) | – | 31.0 (0.4) | – | 6899.8 (92.7) |

| | | | | | | | | | | | | | | | | |
|---|---|---|---|---|---|---|---|---|---|---|---|---|---|---|---|---|
| CRO | 701.3 (6.6) | – | 1.7 (<0.1) | – | 54.5 (0.5) | 895.7 (8.4) | 0.3 (<0.1) | 6.9 (0.1) | 3987.2 (37.4) | 0.6 (<0.1) | 377.3 (3.5) | 0.1 (<0.1) | – | 277.7 (2.6) | – | 4348.0 (40.8) |
| KER | – | – | 1.5 (<0.1) | – | 2.1 (<0.1) | 52.0 (1.0) | – | 1.6 (<0.1) | 1992.9 (38.9) | 0.1 (<0.1) | 58.3 (1.1) | 0.1 (<0.1) | – | 47.0 (0.9) | – | 2963.2 (57.9) |
| AKL | – | 46.6 (1.8) | 62.8 (2.4) | 73.0 (2.8) | 29.4 (1.1) | – | – | 218.5 (8.4) | – | 1950.0 (74.9) | 88.5 (3.4) | 110.7 (4.3) | – | – | 25.3 (1.0) | – |
| ANT | – | 44.6 (2.4) | 1.8 (0.1) | 285.8 (15.3) | 104.0 (5.6) | – | – | 77.6 (4.2) | – | 1080.0 (58.0) | 139.1 (7.5) | 40.9 (2.2) | – | – | 89.6 (4.8) | – |
| FLK | – | 13502.2 (58.7) | – | 392.2 (1.7) | 1561.5 (6.8) | 2579.7 (11.2) | 1213.0 (5.3) | 841.9 (3.7) | – | – | 329.5 (1.4) | 2.3 (<0.1) | 2522.6 (11.0) | – | 42.4 (0.2) | – |
| SOG | – | 16155.7 (63.0) | – | 335.6 (1.3) | 1445.4 (5.6) | 2752.3 (10.7) | 1349.4 (5.3) | 896.5 (3.5) | – | – | 421.6 (1.6) | 21.7 (0.1) | 2251.6 (8.8) | – | 19.4 (0.1) | – |

Populations: PEI = Prince Edward, CRO = Crozet, KER = Kerguelen, AKL = Auckland, ANT = Antipodes, FLK = Falklands, SOG = South Georgia. Flag states: AGO = Angola, ARG = Argentina, AUS = Australia, BRA = Brazil, CHL = Chile, CHN = China, ESP = Spain, FJI = Fiji, FRA = France (including New Caledonia), GBR = United Kingdom of Great Britain and Northern Ireland (including the Falkland Islands), JPN = Japan, KOR = South Korea, NAM = Namibia, NZL = New Zealand, Other = any flag state that did not represent at least 2% of overlap for at least one population, PAN = Panama, PRT = Portugal, SYC = Seychelles, TWN = Taiwan, UKR = Ukraine, URY = Uruguay, VCT = St. Vincent and the Grenadines, VUT = Vanuatu, ZAF = South Africa. The high number of flag states prevented the same flag state featuring for all gear types. – indicates that there was no fleet for given gear type or no overlap with that flag state.

**Table S3** The top ten flag states contributing to overlap of white chinned petrels with pelagic and demersal longline and trawl fisheries based on both logbook and AIS fishing effort data. For each data source and gear type, the top fleets are listed in decreasing order, with the percentage contribution to total overlap (summed across populations but not weighted for population size) in parentheses. Grey-shaded boxes represent flag states contributing to 5% or more of overlap for that gear type.

| Rank | Logbook | | | AIS | | |
|---|---|---|---|---|---|---|
| | Pelagic longline | Demersal longline | Trawl | Pelagic longline | Demersal longline | Trawl |
| 1 | TWN (36.6) | NAM (56.7) | NAM (32.6) | ZAF (29.4) | KOR (27.6) | ARG (39.0) |
| 2 | JPN (32.9) | ZAF (22.6) | ZAF (26.7) | TWN (22.8) | ZAF (24.0) | ZAF (18.6) |
| 3 | ESP (7.0) | FRA (6.7) | ARG (25.3) | JPN (13.6) | CHL (12.0) | NAM (8.4) |
| 4 | ZAF (6.9) | ARG (5.0) | NZL (7.6) | ESP (13.6) | ARG (10.3) | ESP (8.3) |
| 5 | NAM (6.0) | NZL (4.4) | GBR (4.9) | CHN (3.6) | FRA (7.0) | URY (6.3) |
| 6 | KOR (2.7) | GBR (3.4) | URY (2.2) | PRT (2.6) | NAM (5.1) | CHN (4.2) |
| 7 | NZL (2.4) | CHL (0.9) | AUS (0.7) | VUT (2.4) | URY (2.7) | NZL (4.0) |
| 8 | URY (1.1) | AUS (0.2) | CHL (0.1) | NAM (2.4) | GBR (2.6) | GBR (3.4) |
| 9 | FJI (0.9) | JPN (<0.1) | FRA (<0.1) | SYC (2.4) | BRA (2.3) | KOR (2.7) |
| 10 | BRA (0.8) | ESP (<0.1) | RUS (<0.1) | KOR (2.3) | NZL (2.3) | CHL (1.4) |

Flag states: ARG = Argentina, AUS = Australia, BRA = Brazil, CHL = Chile, CHN = China, ESP = Spain, FJI = Fiji, FRA = France (including New Caledonia), GBR = United Kingdom of Great Britain and Northern Island (including the Falkland Islands), JPN = Japan, KOR = South Korea, NAM = Namibia, NZL = New Zealand, PRT = Portugal, RUS = Russia, SYC = Seychelles, TWN = Taiwan, URY = Uruguay, VUT = Vanuatu, ZAF = South Africa.

**Table S4.** The percentage of overlap for each white-chinned petrel population and gear type within Exclusive Economic Zones (EEZ) and in High Seas areas of each Regional Fisheries Management Organisation (RFMO) and the Commission for the Conservation of Antarctic Marine Living Resources (CCAMLR).

| | EEZ | High Seas | | | | |
|---|---|---|---|---|---|---|
| Pelagic longline | | IATTC | ICCAT | IOTC | WCPFC | |
| PEI | 62.9 | 0.0 | 2.9 | 34.2 | 0.0 | |
| CRO | 72.8 | 0.0 | 19.4 | 7.8 | 0.0 | |
| KER | 53.7 | 0.0 | 37.7 | 8.5 | 0.0 | |
| AKL | 19.9 | 75.3 | 0.0 | 0.0 | 4.7 | |
| ANT | 39.6 | 45.9 | 0.0 | 0.2 | 14.3 | |
| FLK | 46.7 | 2.8 | 50.5 | 0.0 | 0.0 | |
| SOG | 33.6 | 6.9 | 59.5 | 0.0 | 0.0 | |
| Demersal longline | | CCAMLR | SEAFO | SIOFA | SPRFMO | No RFMO |
| PEI | 96.9 | 0.2 | <0.1 | 1.5 | 0.0 | 1.4 |
| CRO | 98.1 | 0.1 | <0.1 | 0.6 | 0.0 | 1.1 |
| KER | 98.8 | 0.2 | <0.1 | <0.1 | 0.0 | 1.0 |
| AKL | 93.8 | <0.1 | 0.0 | 0.0 | 0.1 | 6.1 |
| ANT | 89.9 | <0.1 | 0.0 | 0.0 | 0.1 | 10.0 |
| FLK | 63.0 | <0.1 | 0.0 | 0.0 | 0.0 | 37.0 |
| SOG | 51.8 | 0.1 | 0.0 | 0.0 | 0.0 | 48.0 |
| Trawl | | CCAMLR | SEAFO | SIOFA | SPRFMO | No RFMO |
| PEI | 100.0 | 0.1 | <0.1 | <0.1 | 0.0 | <0.1 |
| CRO | 100.0 | <0.1 | <0.1 | <0.1 | 0.0 | <0.1 |
| KER | 100.0 | <0.1 | <0.1 | <0.1 | 0.0 | 0.0 |
| AKL | 96.1 | 0.1 | 0.0 | 0.0 | 3.7 | 0.1 |
| ANT | 85.6 | 0.1 | 0.0 | 0.0 | 13.8 | 0.5 |
| FLK | 98.7 | 0.1 | 0.1 | 0.0 | 0.3 | 0.9 |
| SOG | 98.7 | <0.1 | 0.1 | 0.0 | 0.1 | 1.1 |

Population: PEI = Prince Edward, CRO = Crozet, KER = Kerguelen, AKL = Auckland, ANT = Antipodes, FLK = Falklands, SOG = South Georgia. RFMO: IATTC = Inter-American Tropical Tuna Commission, ICCAT = International Commission for the Conservation of Atlantic Tunas, IOTC = Indian Ocean Tuna Commission, SEAFO = South East Atlantic Fisheries Organisation, SIOFA = Southern Indian Ocean Fisheries Agreement, SPRFMO = South Pacific Regional Fisheries Management Organisation, WCPFC = Western and Central Pacific Fisheries Commission.

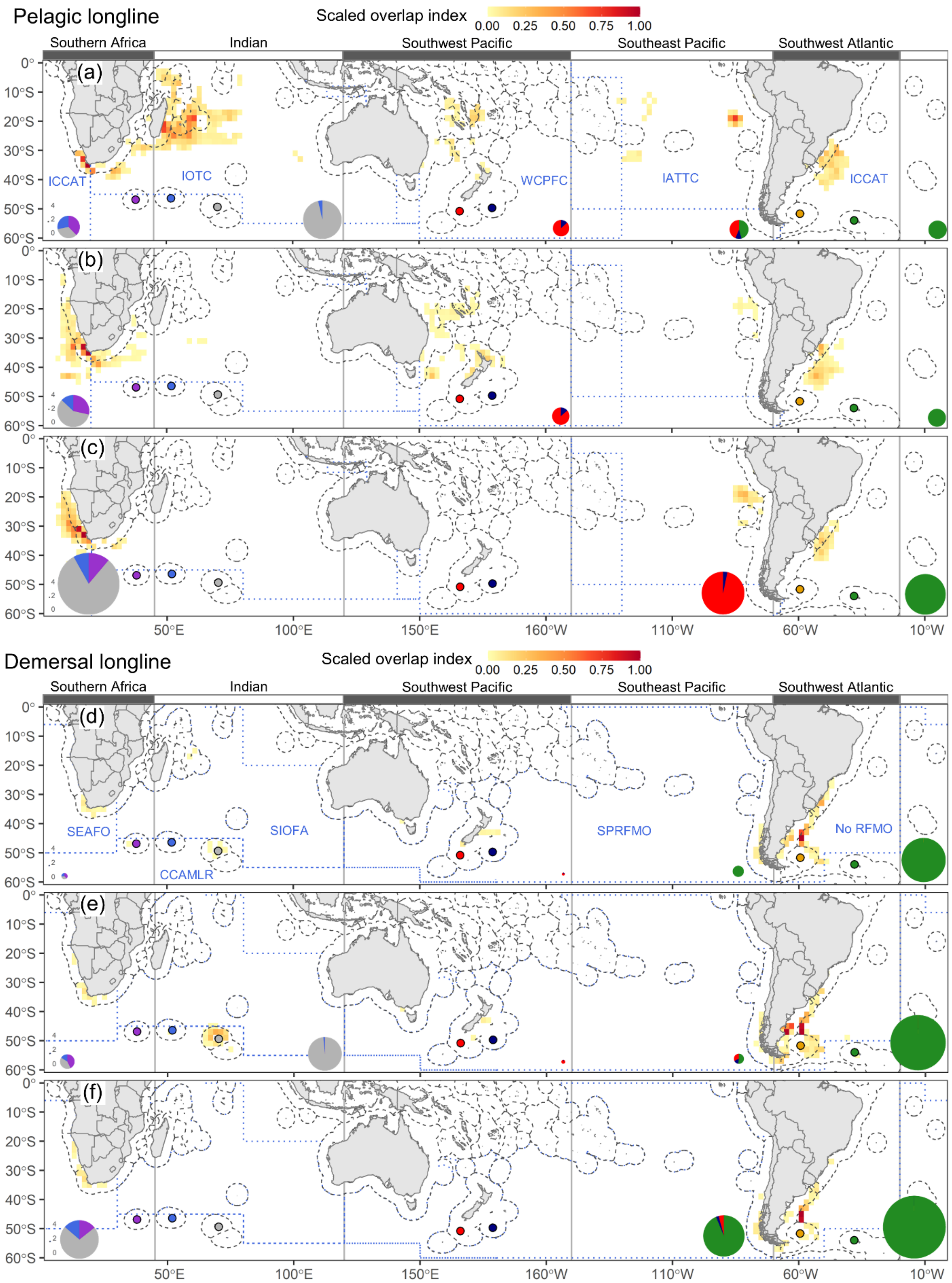

Pelagic longline
Scaled overlap index
0.00 0.25 0.50 0.75 1.00
Southern Africa
Indian
Southwest Pacific
Southeast Pacific
Southwest Atlantic
(a)
(b)
(c)
ICCAT
IOTC
WCPFC
IATTC
ICCAT
0°
10°S
20°S
30°S
40°S
50°S
60°S
50°E
100°E
150°E
160°W
110°W
60°W
10°W
Demersal longline
Scaled overlap index
0.00 0.25 0.50 0.75 1.00
Southern Africa
Indian
Southwest Pacific
Southeast Pacific
Southwest Atlantic
(d)
(e)
(f)
SEAFO
CCAMLR
SIOFA
SPRFMO
No RFMO

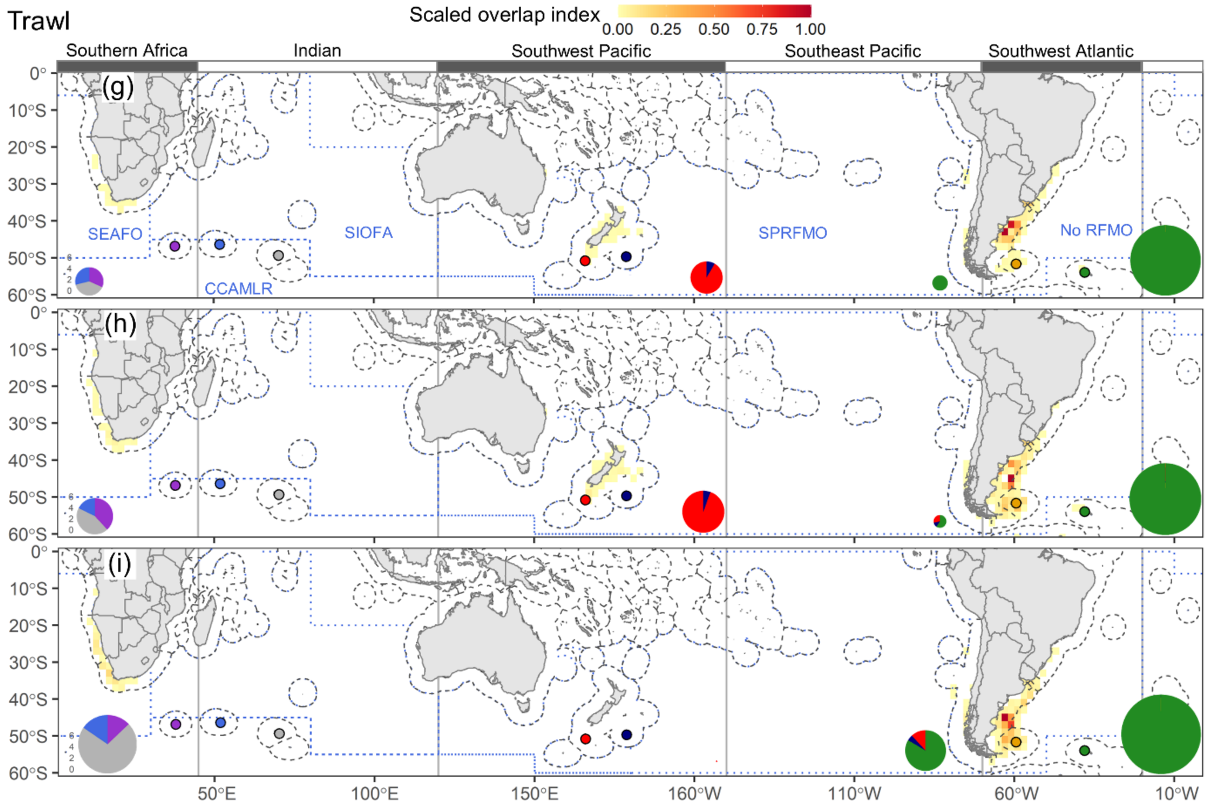


**Figure S1.** Global fisheries-overlap hotspots for white-chinned petrels with a–c) pelagic longline, d–f) demersal longline and g–h) trawl fisheries, and the contribution of each population, during pre-laying (a, d, g; October–November), breeding (b, e, h; December–April) and non-breeding (c, f, i; May–September). Fisheries overlap was calculated for each population and gear type, weighted by population size, summed across gear types and scaled to a maximum index value of 1. Fisheries overlap was divided into five zones (shown by longitudinal grey lines) and the proportion represented by each population is shown by a pie chart, with the size scaled for each region according to the total risk. Numbers on bottom left pie chart indicate the log of the total fisheries overlap score in each region. Boundaries of Regional Fisheries Management Organizations (RFMOs) and the Commission for the Conservation of Antarctic Marine Living Resources (CCAMLR) are shown by dotted blue lines and boundaries of Exclusive Economic Zones are shown by dashed grey lines. IATTC = Inter-American Tropical Tuna Commission, ICCAT = International Commission for the Conservation of Atlantic Tunas, IOTC = Indian Ocean Tuna Commission, SEAFO = South East Atlantic Fisheries Organisation, SIOFA = Southern Indian Ocean Fisheries Agreement, SPRFMO = South Pacific Regional Fisheries Management Organisation, WCPFC = Western and Central Pacific Fisheries Commission.

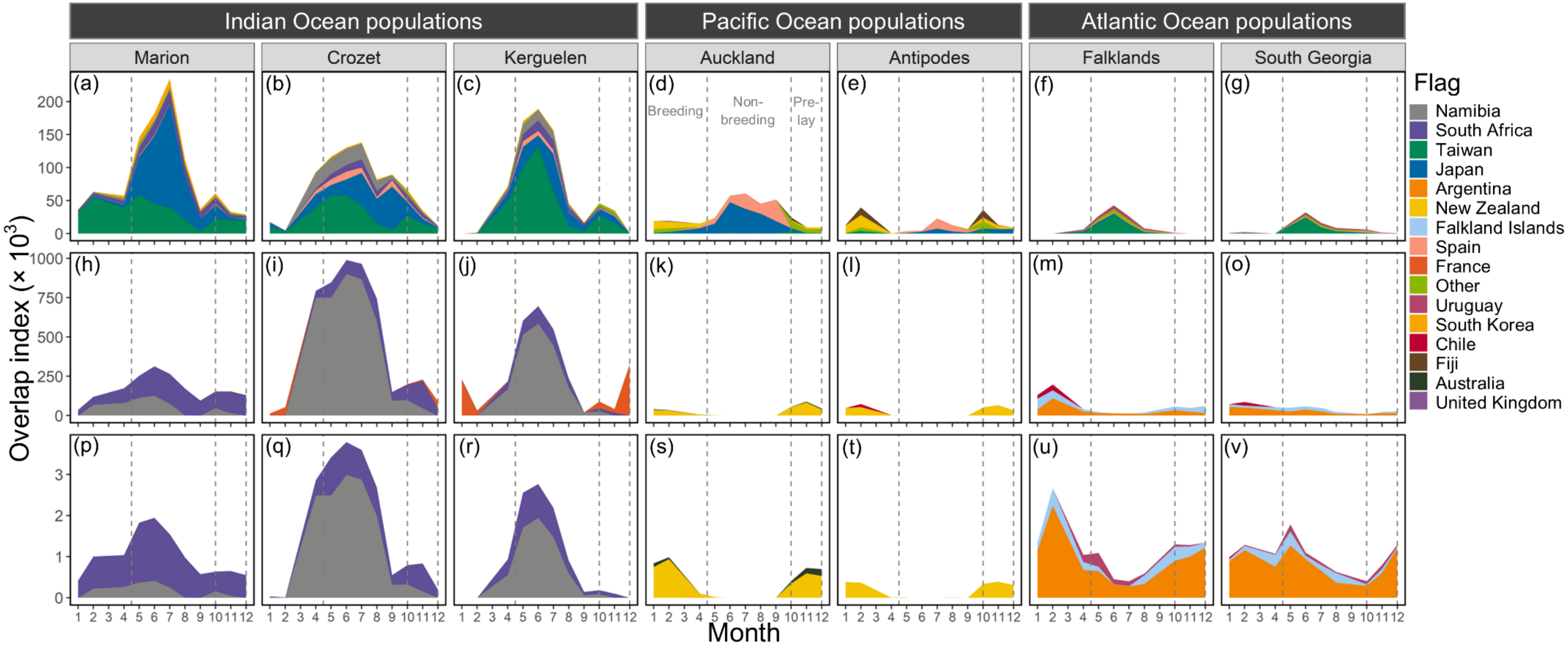


**Figure S2.** Monthly overlap of white-chinned petrel populations with pelagic longline (top row), demersal longline (middle row) and trawl (bottom row) fishing effort, according to flag state. Overlap was conducted with vessel logbooks effo[illegible] 5° spatial resolution. Flag states were assigned the same colour across gear types (rows). Dashed vertical lines indicate the timing of breeding, [illegible]on-breeding and pre-laying periods.

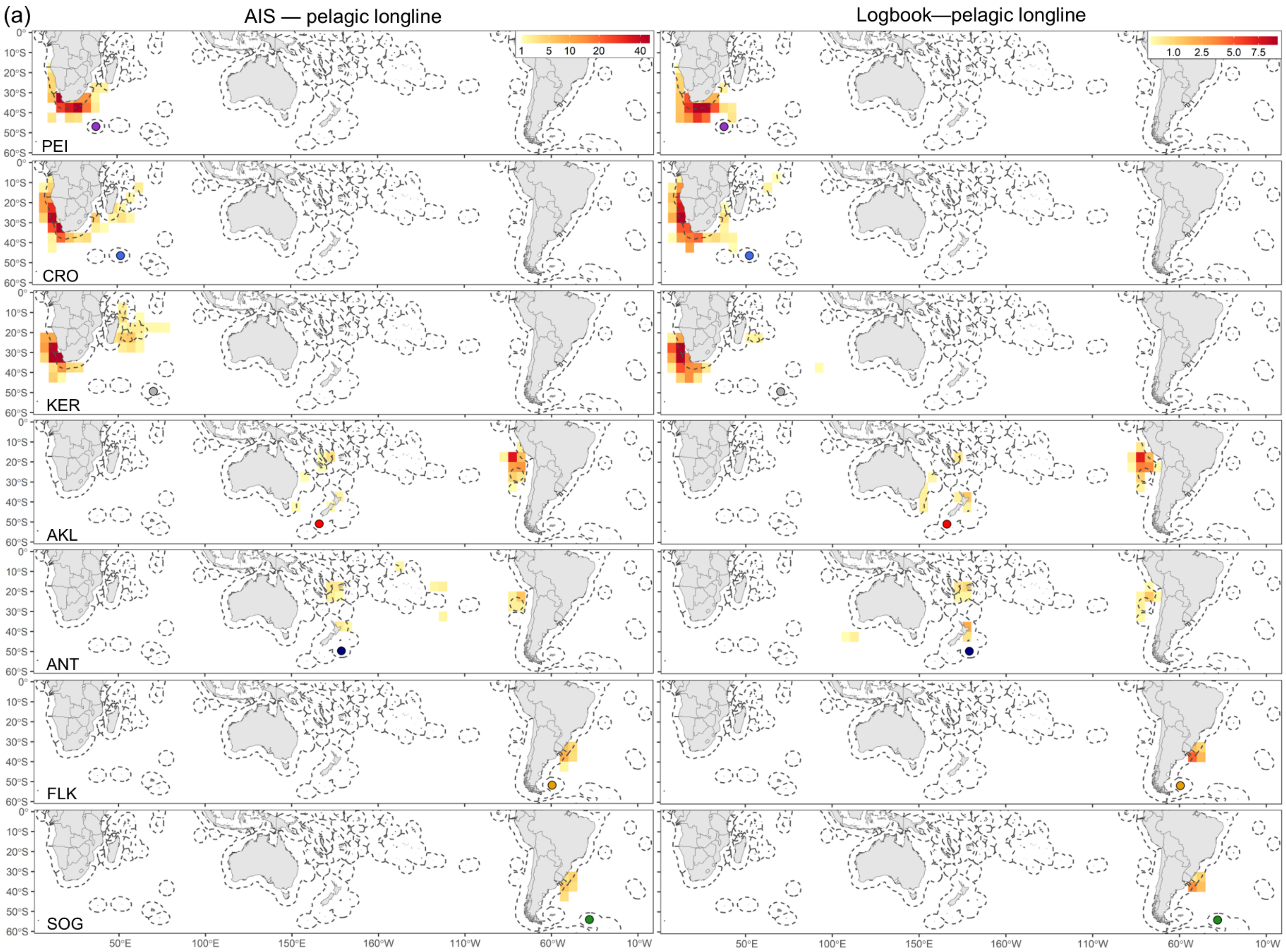
(a)
AIS — pelagic longline
Logbook—pelagic longline
1
5
10
20
40
1.0
2.5
5.0
7.5
PEI
CRO
KER
AKL
ANT
FLK
SOG
0°
10°S
20°S
30°S
40°S
50°S
60°S
50°E
100°E
150°E
160°W
110°W
60°W
10°W

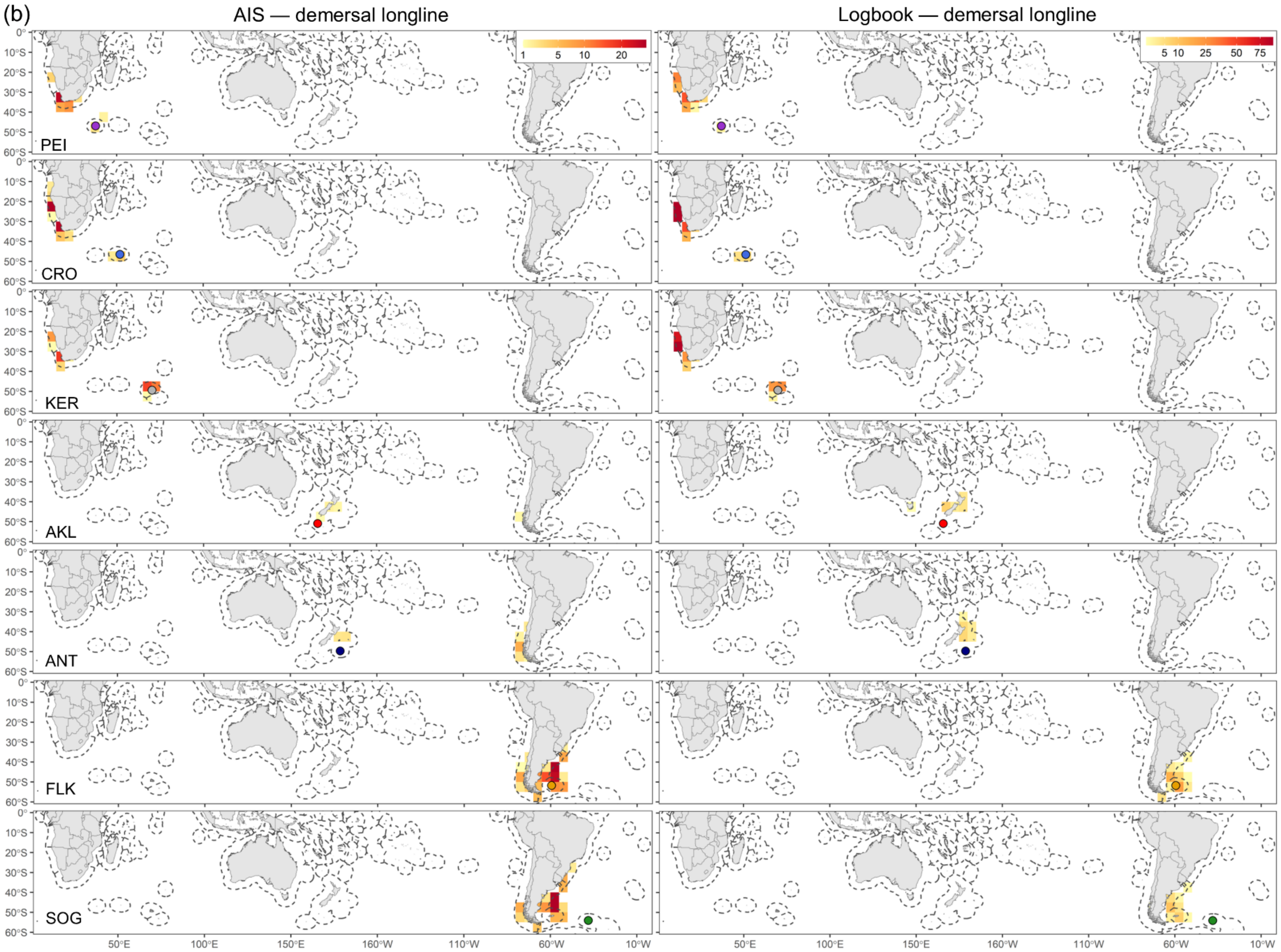
(b)
AIS — demersal longline
Logbook — demersal longline
1 5 10 20
5 10 25 50 75
PEI
CRO
KER
AKL
ANT
FLK
SOG
0°
10°S
20°S
30°S
40°S
50°S
60°S
50°E
100°E
150°E
160°W
110°W
60°W
10°W

(c)

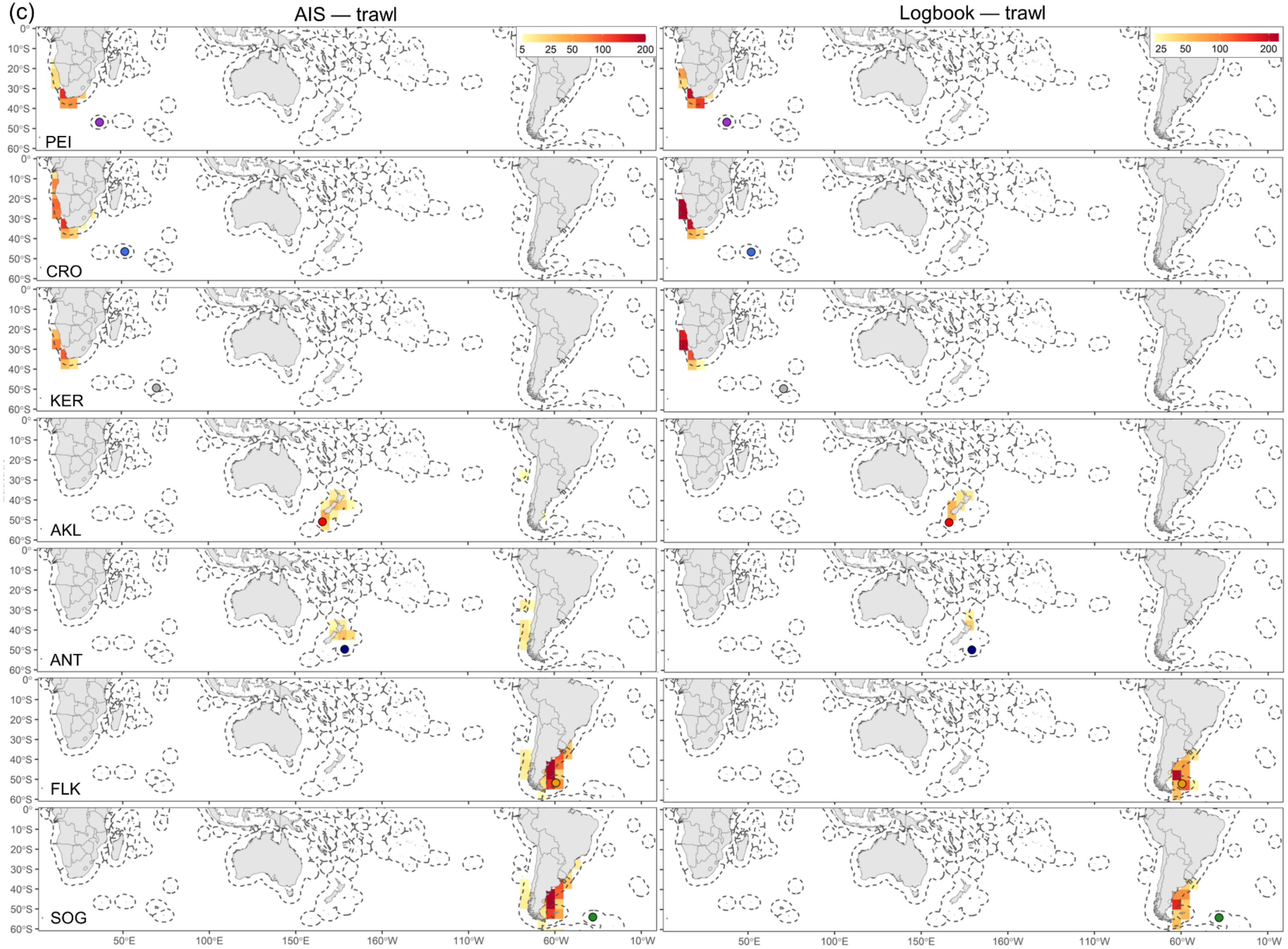
AIS — trawl
Logbook — trawl
5 25 50 100 200
25 50 100 200
0°
10°S
20°S
30°S
40°S
50°S
60°S
PEI
CRO
KER
AKL
ANT
FLK
SOG
50°E
100°E
150°E
160°W
110°W
60°W
10°W

**Figure S3.** Maps of fisheries overlap for each white-chinned petrel population and fishing effort data source (Automatic Identification System [AIS] and logbook) for a) pelagic longline, b) demersal longline and c) trawl fisheries. Cells are coloured according to fisheries-overlap score (plotted on a square root scale) and only the top 25% of overlap values (identified as high-risk zones) are shown. To reduce the influence of a few extremely high values, any value greater than the top 1% was set to that reference value. Overlap values were averaged across all months and those based logbook longline data are divided by $10^3$. PEI = Prince Edward, CRO = Crozet, KER = Kerguelen, AKL = Auckland, ANT = Antipodes, FLK = Falklands and SOG = South Georgia.
