# Supplementary material for: Metapopulation distribution shapes year‐round overlap with fisheries for a circumpolar seabird
Source: Ecol Appl. 2025 Apr 21;35(3):e70019. doi: 10.1002/eap.70019 (PMC12010314; doi:10.1002/eap.70019)
Supplement: Supplementary file 4 — Appendix S4: [file EAP-35-e70019-s003.pdf]

Supporting Information. Kalinka Rexer-Huber, Thomas A. Clay, Paulo Catry, Igor Debski, Graham Parker, Raül Ramos, Bruce C. Robertson, Peter G. Ryan, Paul M. Sagar, Andrew Stanworth, David R. Thompson, Geoffrey N. Tuck, Henri Weimerskirch. Richard A. Phillips. 2025. Metapopulation distribution shapes year-round overlap with fisheries for a circumpolar seabird. Ecological Applications.

Appendix S4: Comparing monthly overlap and captures of white-chinned petrels in New Zealand fisheries

To examine whether our metric of fisheries overlap matched seasonal trends in white-chinned petrel (*Procellaria aequinoctialis*) bycatch, we focussed on New Zealand fisheries, given that bycatch data are freely accessible online. Data on protected species bycatch were downloaded from the Ministry of Primary Industries (accessed from https://protectedspeciescaptures.nz on 07/07/2022). From 2002 to 2020, white-chinned petrels were captured in the trawl fishery mostly targeting arrow squid (*Nototodarus* spp.), hoki (*Macruronus novaezelandiae*), silver warehou (*Seriolella punctata*), New Zealand scampi (*Metanephrops challenger*), barracouta (*Thyrsites atun*), ling (*Genypterus blacodes*) and jack mackerel (*Trachurus* spp.) (*n* = 2,418 birds), the demersal longline fishery mostly targeting ling and ribaldo (*Mora moro*) (*n* = 492 birds), and the pelagic longline fishery targeting southern bluefin (*Thunnus maccoyii*), bigeye (*T. obesus* and albacore (*T. alalunga*) tunas and broadbill swordfish (*Xiphias gladius*) (*n* = 49). Observers were only present on some vessels and observer coverage varied depending on the fishery: 5.3–23.6% for trawl (depending on the year; mean ± SD: 12.6 ± 5.6%), 2.2–29.3% for demersal longline (8.9 ± 5.7%) and 9.4–30.7% (20.5 ± 6.5%) for pelagic longline fisheries. There are also likely to be additional cryptic mortalities not recorded by observers and so we acknowledge that captures are likely to be substantially higher than those observed.

We plotted observed monthly captures in relation to fisheries-overlap scores for birds from Auckland and Antipodes populations (Appendix S4: Fig. S1), given they were the only tracked populations that encountered New Zealand fisheries. Overlap with fisheries and observed captures were both generally highest during the austral summer (October–April). For demersal longline and trawl fisheries, there were two peaks in petrel captures in October–November and February–April which were reflected in fisheries-overlap scores; however, overlap scores appeared to underestimate actual bycatch risk from demersal longline fisheries in February–April and overestimate risk from trawl fisheries in October–December (Appendix S4: Fig. S1b, c). In contrast, most observed captures by pelagic longline fisheries occurred during April and May, when predicted overlap with fisheries was low (Appendix S4: Fig S1a). We note that these correlations are simplistic and do not account for several factors affecting bycatch rates, such as variable observer coverage, cryptic mortality and use of bycatch mitigation, which we assumed to be fairly even across the year, as well as the lack of tracking data from Campbell birds. Regardless, our coarse-scale analysis of fisheries overlap (representing potential bycatch risk) broadly captured the periods of highest actual bycatch risk for white-chinned petrels from demersal longline and trawl, but not pelagic longline fisheries.

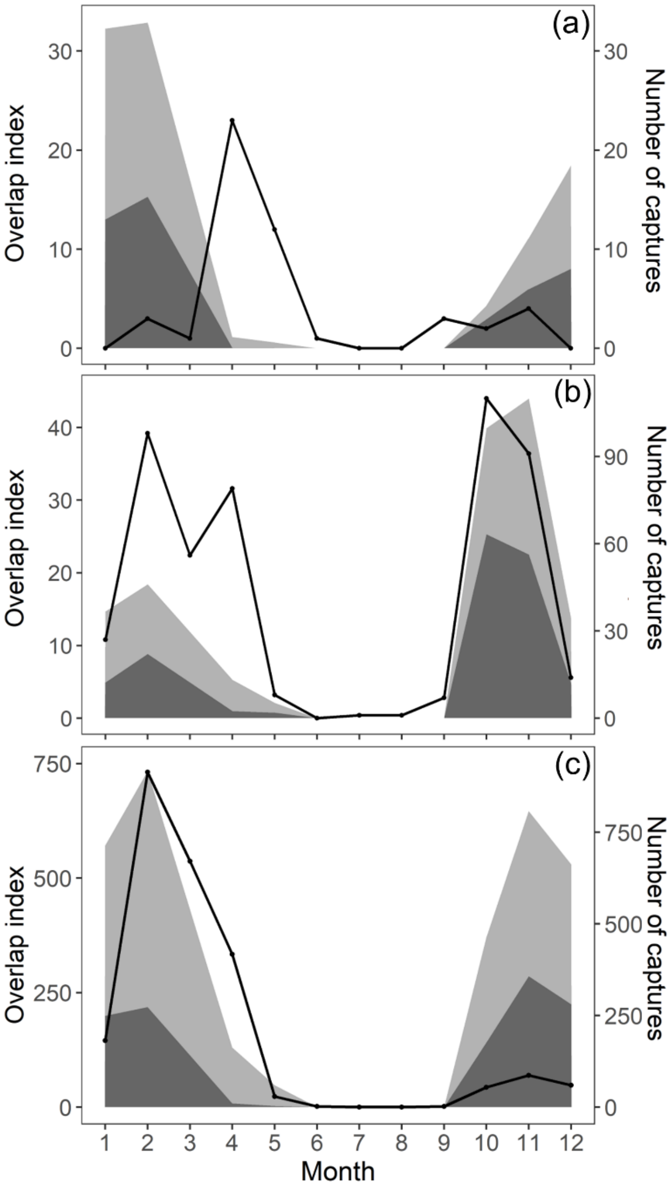

Figure S1. Monthly observed captures of white-chinned petrels (*Procellaria aequinoctialis*) and overlap with New Zealand a) pelagic longline, b) demersal longline and c) trawl fisheries. Captures are indicated by the black line and were recorded during 2002–2020. Monthly fisheries overlap for the Auckland (light grey shading) and Antipodes (dark grey shading) island populations are based on Automatic Identification System (AIS) data during 2012–2020 and are stacked to show total overlap for the two populations.
